# Supplementary material for: Structural basis of dimerization of chemokine receptors CCR5 and CXCR4
Source: Nat Commun. 2023 Oct 13;14:6439. doi: 10.1038/s41467-023-42082-z (PMC10575954; doi:10.1038/s41467-023-42082-z)
Supplement: Supplementary file 1 — Supplementary Information [file 41467_2023_42082_MOESM1_ESM.pdf]

Supplementary Information

Structural basis of dimerization of chemokine receptors CCR5 and CXCR4

Daniele Di Marino[#,1,2,3], Paolo Conflitti[#,4], Stefano Motta[#,5] & Vittorio Limongelli[*,4]

[1]*Department of Life and Environmental Sciences - New York-Marche Structural Biology Centre (NY-MaSBiC), Polytechnic University of Marche, Via Brecce Bianche, 60131 Ancona, Italy.*

[2]*Neuronal Death and Neuroprotection Unit, Department of Neuroscience, Mario Negri Institute for Pharmacological Research-IRCCS, Via Mario Negri 2, 20156 Milano, Italy*

[3]*National Biodiversity Future Center (NBFC), Palermo, Italy*

[4]*Università della Svizzera italiana (USI), Faculty of Biomedical Sciences, Euler Institute, Via G. Buffi 13, CH-6900 Lugano, Switzerland.*

[5]*Department of Earth and Environmental Sciences, University of Milano-Bicocca, Piazza della Scienza 1, 20126 Milan, Italy.*

[*]Corresponding author: Prof. Dr. Vittorio Limongelli, email: vittoriolimongelli@gmail.com or vittorio.limongelli@usi.ch

[#]These authors contributed equally to the work

This document includes:

Supplementary Tables 1-6

Supplementary Figures 1-19

Supplementary Discussion: The role of cholesterol in the dimerization process

Supplementary Discussion: Dimerisation mechanism

Supplementary Methods

Supplementary References

Supplementary Table 1: Number of binding and unbinding events for each system.

CCR5-CCR5	566
CXCR4-CXCR4	146
CCR5-CXCR4	327

Supplementary Table 2: Definition of the free energy minima obtained from Coarse-Grained MetaDynamics (CG-MetaD) calculations as intervals of the distance (r) and torsion (Ω) CVs.

		Distance (nm)	Torsion (rad)
CCR5-CCR5	Basin A	3.1 – 3.3	-1.0 – -0.4
	Basin B	3.4 – 3.8	-2.7 – -2.2
CXCR4-CXCR4	Basin A	2.6 – 2.7	-1.3 – -1.8
	Basin B	2.7 – 2.9	0.2 – 0.7
CCR5-CXCR4	Basin A	2.8 – 3.1	-0.8 – -0.4
	Basin B	3.4 – 3.7	-2.8 – -2.2

Supplementary Table 3. Lipidic composition of the realistic model (also defined as the plasma membrane) of cellular membrane used in (a) Martini 2, (b) Martini 3, and (c) atomistic simulations. The parameters for some lipids are missing in the Martini 3 and atomistic force fields, therefore these were not included in the plasma membrane model. Table legend: PC = phosphatidylcholines; POPC = 1-palmitoyl-2-oleoyl-sn-glycero-3-phosphatidylcholines; DOPC = 1,2-dioleoyl-sn-glycero-3-phosphatidylcholines; PE = phosphatidylethanolamine; POPE = 1-palmitoyl-2-oleoyl-sn-glycero-3-phosphoethanolamine; DOPE = 1,2-dioleoyl-sn-glycero-3-phosphoethanolamine; Sph = sphingolipid; POSM = N-(9Z-octadecenoyl)-hexadecasphing-4-enine-1-phosphocholine; GM3 = monosialodihexosylganglioside; DPG3 = neuAcalpha2-3Galbeta1-4Glcbeta-Cer(d16:1/16:0); Chol = cholesterol; PS = phosphatidylserine; POPS = 1-hexadecanoyl-2-(9Z-octadecenoyl)-sn-glycero-3-phosphoserine; DOPS = 1,2-di-(9Z-octadecenoyl)-sn-glycero-3-phosphoserine; PIP2 = phosphatidylinositol 4,5-bisphosphate; DPP2 = CG model corresponding to the atomistic C16:0 dipalmitoyl phosphatidylinositol 4,5-bisphosphate (DP-PIP2) – C18:0 distearoyl phosphatidylinositol 4,5-bisphosphate (DS-PIP2).

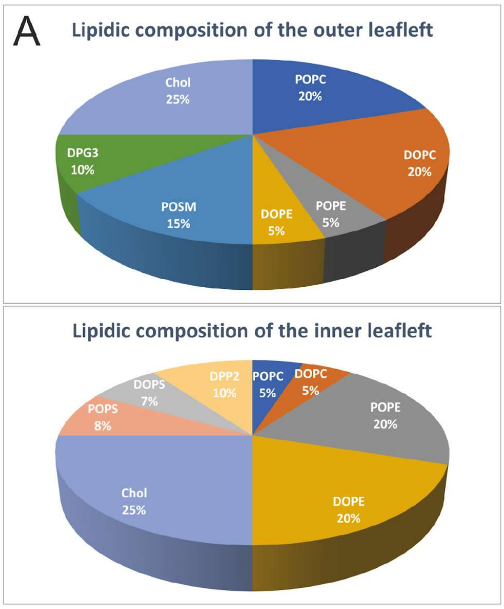

Lipid Family	Molecule	% Outer Leaflet	% Inner Leaflet
PC	POPC	20	5
	DOPC	20	5
PE	POPE	5	20
	DOPE	5	20
Sph	POSM	15	
GM3	DPG3	10	
Chol	Chol	25	25
PS	POPS		8
	DOPS		7
PIP2	DPP2		10

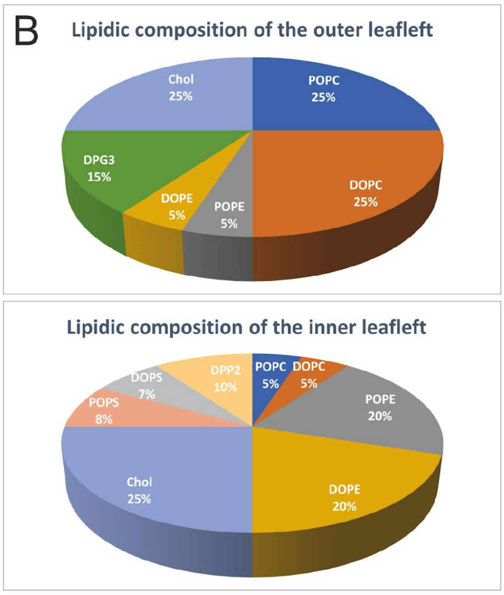

Lipid Family	Molecule	% Outer Leaflet	% Inner Leaflet
PC	POPC	25	5
	DOPC	25	5
PE	POPE	5	20
	DOPE	5	20
GM3	DPG3	15	
Chol	Chol	25	25
PS	POPS		8
	DOPS		7
PIP2	DPP2		10

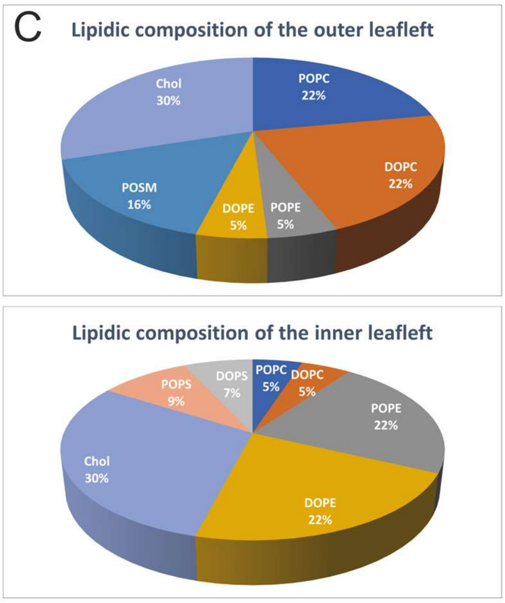

Lipid Family	Molecule	% Outer Leaflet	% Inner Leaflet
PC	POPC	22	5
	DOPC	22	5
PE	POPE	5	22
	DOPE	5	22
Sph	POSM	16	
Chol	Chol	30	30
PS	POPS		9
	DOPS		7

Supplementary Table 4. Results of the in-silico mutagenesis experiments performed using the atomistic structures of CCR5 and CXCR4 homodimers back mapped from the minima identified via CG-MetaD and the Mutabind2 and mCSC-PPI2 webservers. Mutations are reported using the one-letter notation. Superscripts refer to the Ballesteros-Weinstein numbering scheme[1].

				ΔΔG	
System	Minimum	Protomer	Mutations	MutaBind2	mCSC-PPI2
CCR5 - CCR5	M1	A	L196$^{5.40}$K	-0.84	-0.25
			I200$^{5.44}$K	-0.89	-0.689
			L205$^{5.49}$K	-1.26	-0.786
		B	V150$^{4.47}$A	-0.89	0.187
			L196$^{5.40}$K	-0.91	-0.26
			I200$^{5.44}$K	-0.94	-0.473
			L205$^{5.49}$K	-0.9	-0.231
	M2	A	L196$^{5.40}$K	-0.82	-0.708
			I200$^{5.44}$K	-1.39	-0.811
			L205$^{5.49}$K	-1.08	-0.656
		B	I52$^{1.54}$V	-0.22	-0.182
	4MBS	B	V150A	-0.08	-0.09
CXCR4 - CXCR4	M1	A	K239$^{6.35}$E	-0.16	-0.964
			L246$^{6.42}$A	-0.73	-0.14
			L246$^{6.42}$P	-1.48	-0.748
		B	K239$^{6.35}$E	-0.35	-0.772
	M2	B	K239$^{6.35}$E	-1.68	-0.961
			V242$^{6.38}$D	-0.97	-0.067
			V242$^{6.38}$A	-0.56	0.201
			L246$^{6.42}$A	-2.08	-1.217
			L246$^{6.42}$P	-2	-1.274
	3OE8	B	K239$^{6.35}$E	-0.2	-0.289
			V242$^{6.38}$D	-0.54	-0.061
			V242$^{6.38}$A	-0.33	-0.123
	3OE9	N/A	N/A	N/A	N/A

Supplementary Table 5. Results of the alanine scan calculations performed using the atomistic structures of CCR5 and CXCR4 homodimers and CCR5-CXCR5 heterodimers back mapped from the minima identified via CG-MetaD and the Mutabind2 and mCSC-PPI2 webservers. Only the single point mutations contributing to a ΔΔG higher than 1 kcal/mol are reported. Mutations are reported using the one-letter notation. Superscripts refer to the Ballesteros-Weinstein numbering scheme[1].

CCR5 – CCR5				ΔΔG	
System	Minimum	Protomer	Mutations	MutaBind2	mCSC-PPI2
CCR5 - CCR5	M1	A	F135^{ICL2}A	-2.32	-1.31
			F158$^{4.55}$A	-1.64	-1.13
			L161$^{4.58}$A	-1.78	-1.32
			P162$^{4.59}$A	-2.19	-1.65
			I165$^{4.62}$A	-2.57	-1.24
			F166$^{4.63}$A	-1.95	-1.4
			L201$^{5.45}$A	-1.97	-1.03
		B	F158$^{4.55}$A	-3.36	-1.9
			P162$^{4.59}$A	-1.44	-1.22
			I165$^{4.62}$A	-2.58	-1.29
			F166$^{4.63}$A	-2.35	-1.71
			W190$^{5.34}$A	-2.43	-1.22
	M2	A	F117$^{3.41}$A	-1.33	-1.3
			L128$^{3.52}$A	-1.42	-1.33
			F144$^{4.41}$A	-1.64	-1.6
			F158$^{4.55}$A	-2.45	-1.35
			W190$^{5.34}$A	-2	-1.61
		B	F41$^{1.43}$A	-1.33	-1.29
			F45$^{1.47}$A	-3.17	-1.84
			L53$^{1.55}$A	-2.44	-1.48
			I56$^{1.58}$A	-1.16	-1.16
			Y58$^{1.60}$A	-1.48	-1.64
			F85$^{2.59}$A	-2.12	-1.83
			H88$^{2.62}$A	-1.47	-1.22
			F96^{ECL1}A	-1.64	-1.13
CXCR4 – CXCR4				ΔΔG	
System	Minimum	Protomer	Mutations	MutaBind2	mCSC-PPI2
CXCR4 - CXCR4	M1	A	F36$^{1.30}$A	-1.67	-1.33
			F40$^{1.34}$A	-1.33	-1.32
			W283$^{7.34}$A	-1.25	-1.32
			L297$^{7.48}$A	-1.51	-1.33
			I300$^{7.51}$A	-2.53	-1.34
			L301$^{7.52}$A	-1.07	-1.58

		B	F36$^{1.30}$A	-2.35	-1.27
			F40$^{1.34}$A	-1.83	-1.07
			W283$^{7.34}$A	-1.83	-1.18
			I300$^{7.51}$A	-1.1	-1.28
			L301$^{7.52}$A	-2.56	-1.56
	M2	A	L132$^{3.48}$A	-1.61	-1.17
			Y135$^{3.5}$1A	-1.84	-1.37
			L136$^{3.52}$A	-1.63	-1.2
			P163$^{4.52}$A	-2.05	-1.74
			L167$^{4.56}$A	-2.46	-1.49
			I169$^{4.58}$A	-2.24	-1.18
			P170$^{4.59}$A	-1.67	-1.27
			I173$^{4.62}$A	-1.3	-1.04
			W195$^{5.34}$A	-1.77	-2.21
			F199$^{5.38}$A	-1.4	-1.73
			V206$^{5.45}$A	-1.51	-1.61
		B	Y219$^{5.58}$A	-1.56	-1.08
			I223$^{5.62}$A	-2.66	-1.08
			L226$^{5.65}$A	-1.5	-1.15
			I243$^{6.39}$A	-2.23	-1.42
			L246$^{6.42}$A	-2.08	-1.22
			L253$^{6.49}$A	-1.77	-1.04
			P254$^{6.50}$A	-1.36	-1.3
			I257$^{6.53}$A	-2	-1.37
			W283$^{7.34}$A	-3.23	-1.86
			I286$^{7.37}$A	-1.7	-1.48
			L290$^{7.41}$A	-2.12	-1.19
CCR5 – CXCR4				ΔΔG	
System	Minimum	Protomer	Mutations	MutaBind2	mCSC-PPI2
CCR5 - CXCR4	M1	A	F41$^{1.43}$A	-1.88	-1.53
			I42$^{1.44}$A	-2.35	-1.16
		B	W283$^{7.34}$A	-1.6	-1.04
			I286$^{7.37}$A	-2.69	-1.29
	M2	A	W190$^{5.34}$A	-1.94	-1.95
			F193$^{5.37}$A	-1.87	-1.68
			L196$^{5.40}$A	-1.44	-1.21
			F263$^{6.63}$A	-1.41	-1.09
		B	P170$^{4.59}$A	-2.48	-1.08
			F174$^{4.63}$A	-1.03	-1.48
			W195$^{5.34}$A	-2.58	-2.09

Supplementary Table 6. Overview of the simulations performed and related simulation time, excluding thermalization and equilibration times, or simulation time needed to setup and test the CG models of the receptors. In the table, "POPC/CHOL" refers to the POPC/CHOL 90:10 membrane model (POPC = 1-palmitoyl-2-oleoyl-sn-glycero-3-phosphatidylcholines, CHOL = cholesterol); "Plasma Membrane" to the realistic bilayer model used in Martini 2 CG-MD simulations whose composition is reported in Supplementary Table 3A; "M3 Plasma Membrane" refers to the realistic bilayer model used in Martini 3 CG-MD and CG-MetaD simulations, whose composition is shown in Supplementary Table 3B; "AA Plasma Membrane" is the realistic bilayer model used in the atomistic simulations whole composition is reported in Supplementary Table 3C.

System Simulated	Membrane	Approach	# Replicas	Simulation time	Total time
CCR5-CCR5	POPC/CHOL	CG MetaD	1	1.4 ms	1.4 ms
CXCR4-CXCR4	POPC/CHOL	CG MetaD	1	2.5 ms	2.5 ms
CCR5-CXCR4	POPC/CHOL	CG MetaD	1	1.6 ms	1.6 ms
CCR5-CCR5 minima	POPC/CHOL	CG-MD	2	50 µs	100 µs
CXCR4-CXCR4 minima	POPC/CHOL	CG-MD	2	50 µs	100 µs
CCR5-CXCR4 minima	POPC/CHOL	CG-MD	2	50 µs	100 µs
CCR5-CCR5 minima	POPC/CHOL	AA-MD	2	0.5 µs	1 µs
CCR5-CCR5 minima	AA Plasma Membrane	AA-MD	2	2 µs	4 µs
CXCR4-CXCR4 minima	POPC/CHOL	AA-MD	2	0.5 µs	1 µs
CXCR4-CXCR4 minima	AA Plasma Membrane	AA-MD	2	2 µs	4 µs
CCR5-CXCR4 minima	POPC/CHOL	AA-MD	2	0.5 µs	1 µs
CCR5-CXCR4 minima	AA Plasma Membrane	AA-MD	2	2 µs	4 µs
CCR5-CCR5	Plasma membrane	CG-MD	32	3.5 µs	112 µs
CCR5-CCR5	M3 Plasma membrane	CG-MD	8	4 µs	32 µs
CCR5-CCR5	M3 Plasma membrane	CG MetaD	8	4 µs	32 µs
CXCR4-CXCR4	Plasma membrane	CG-MD	32	3.5 µs	112 µs
CCR5-CXCR4	Plasma membrane	CG-MD	32	3.5 µs	112 µs
CCR5 G protein-CCR5	Plasma membrane	CG-MD	32	3.5 µs	112 µs
CCR5 G protein-CXCR4	Plasma membrane	CG-MD	32	3.5 µs	112 µs
4MBS crystal	Plasma membrane	CG-MD	8	20 µs	160 µs
4MBS crystal	POPC/CHOL	CG-MD	8	20 µs	160 µs

3OE8 crystal	Plasma membrane	CG-MD	8	20 µs	160 µs
3OE8 crystal	POPC/CHOL	CG-MD	8	20 µs	160 µs
3OE9 crystal	Plasma membrane	CG-MD	8	20 µs	160 µs
3OE9 crystal	POPC/CHOL	CG-MD	8	20 µs	160 µs

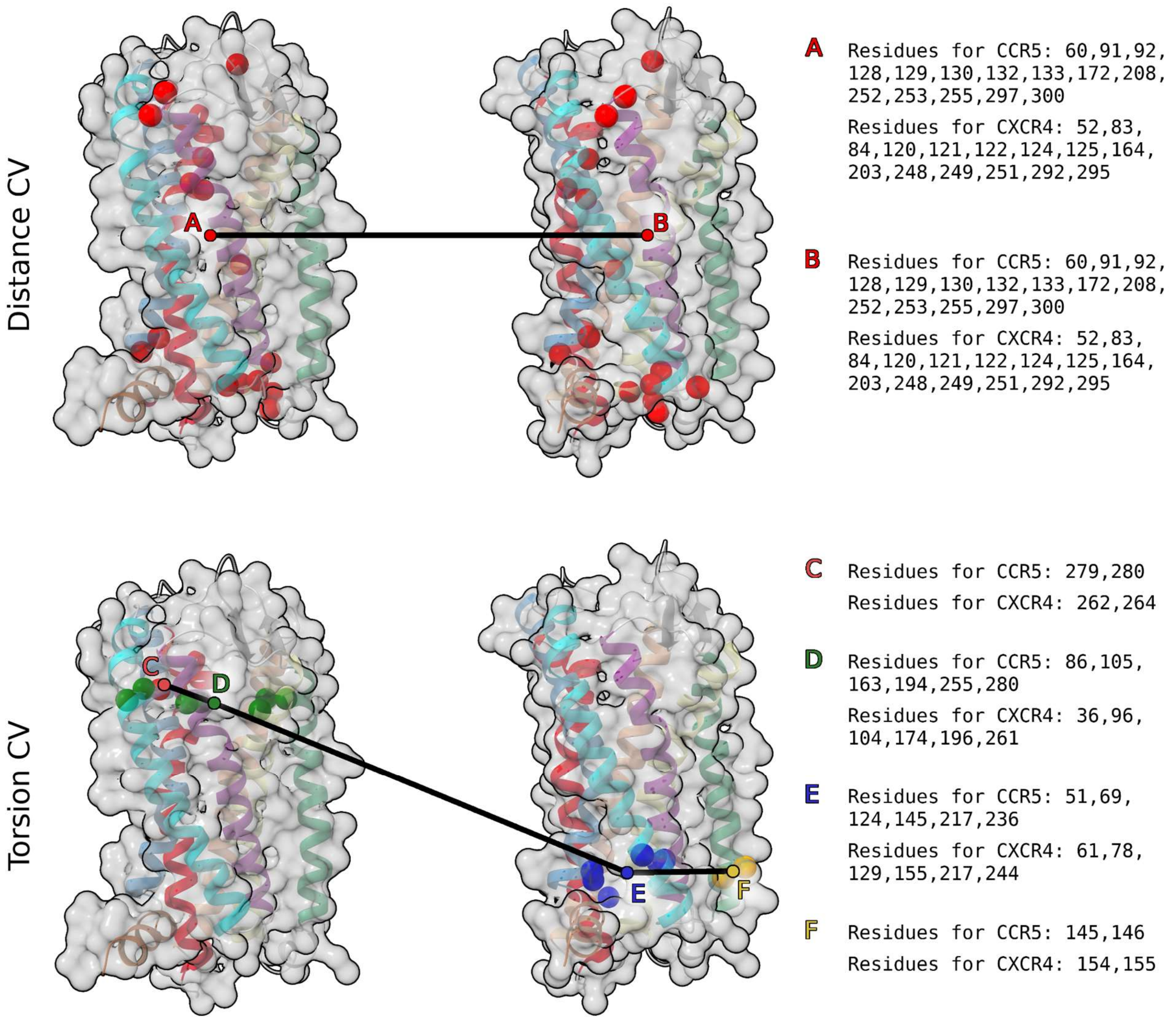

Supplementary Figure 1. **Definition of the Collective Variables (CVs).** The centre of mass of the CG backbone beads (BB) of the residues indicated in the figure was used to define the distance and torsion CVs. As regards CCR5-CXCR4 heterodimer, the A, C and D points were used for CCR5, while the B, E and F points were used for CXCR4.

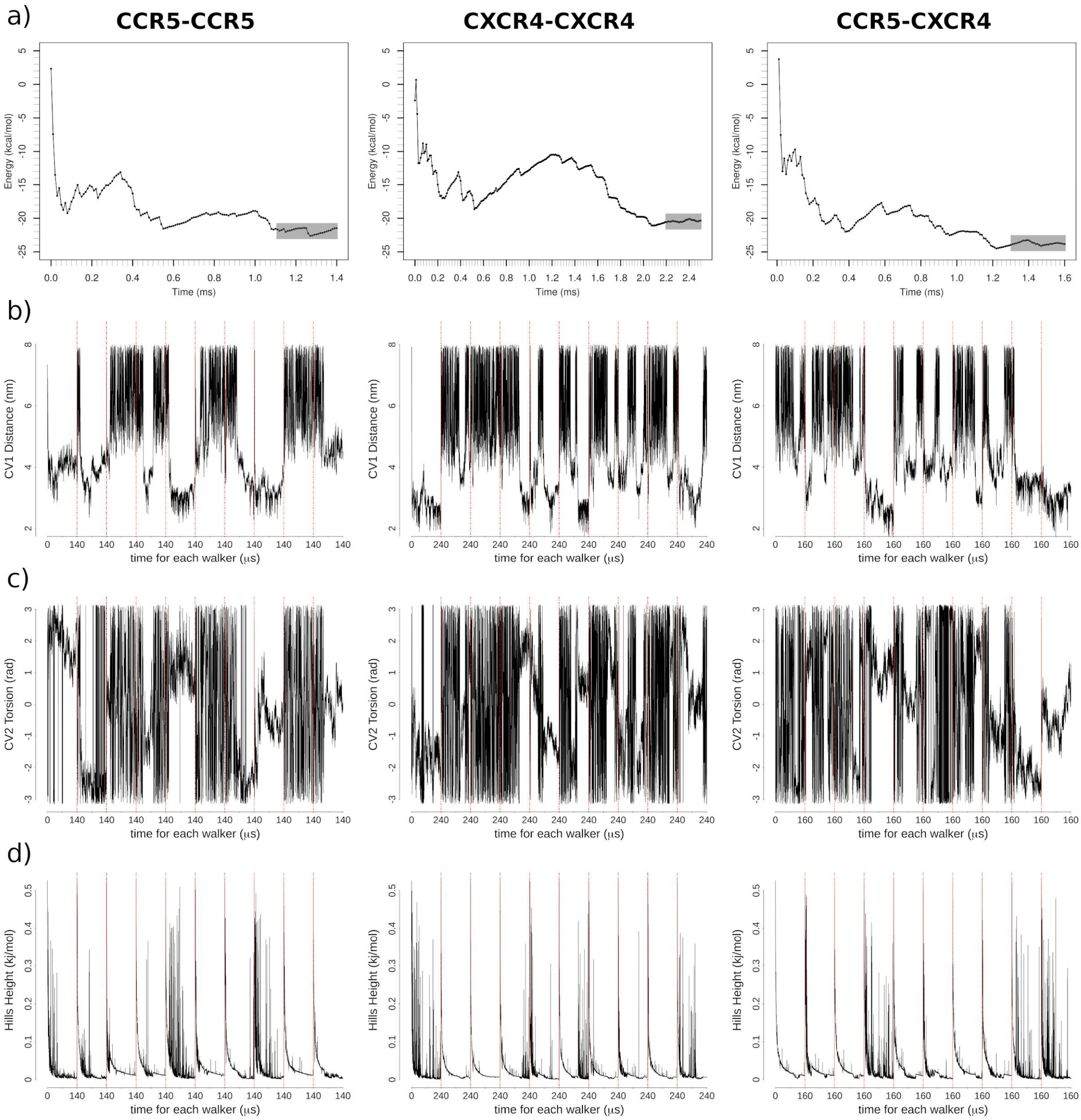

Supplementary Figure 2. **Evolution of CG-MetaD simulations. a** Evolution of the free-energy difference between bound and unbound state as a function of the simulation time. In the last 0.3 ms of simulation, highlighted in grey, the calculations converged leading to absolute binding free-energy estimates of -22.2 (+/- 0.3) kcal/mol for CCR5 homodimer, -21.1 (+/- 0.2) kcal/mol for CXCR4 homodimer, and -24.5 (+/- 0.2) kcal/mol for CCR5-CXCR4 heterodimer. **b** Plots of the distance CV, **c** torsion CV and **d** hills height during the multiple walker CG-MetaD calculations.

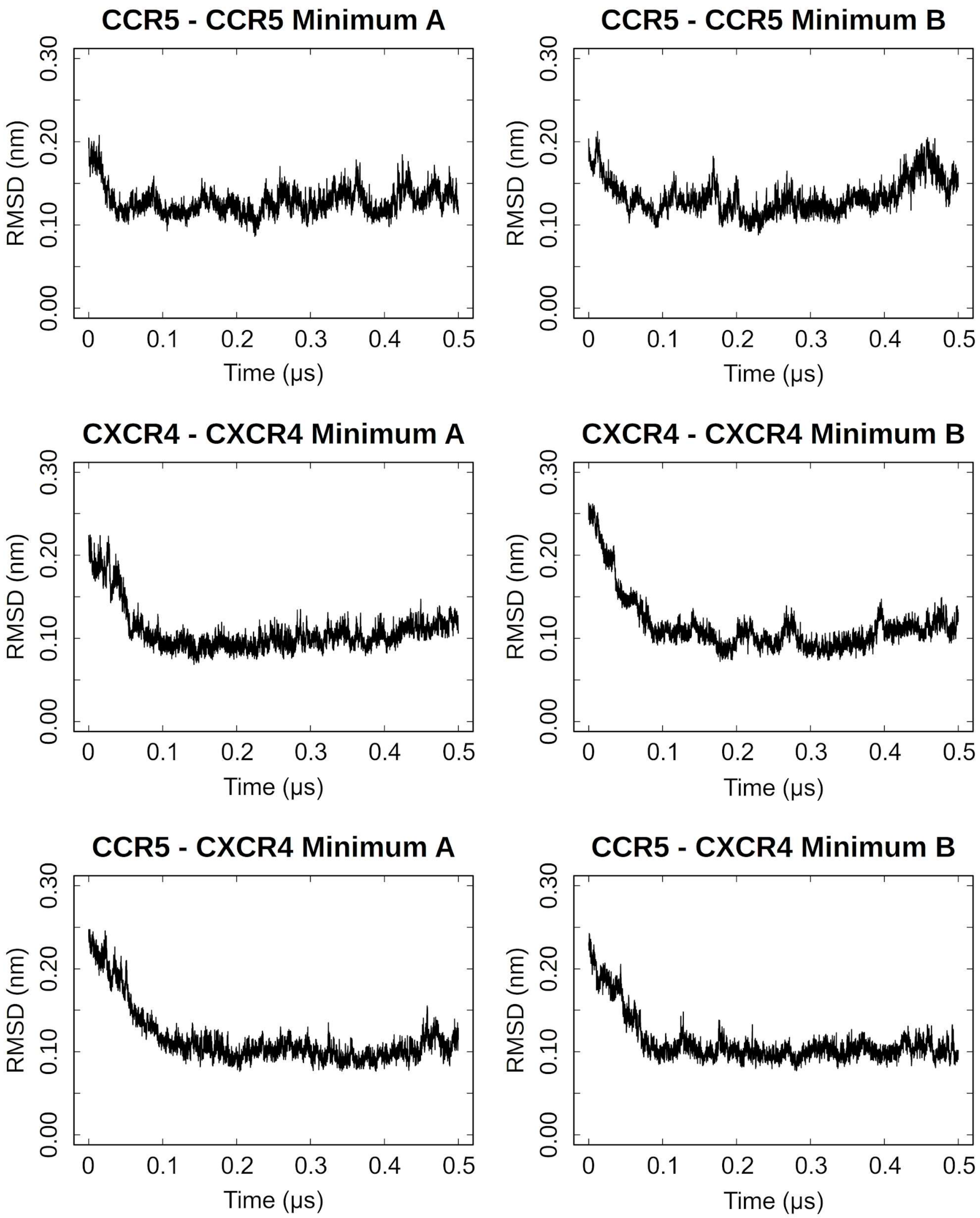

Supplementary Figure 3. **Stability of the identified minima in the simple membrane model.** Plots of the Root Mean Square Deviation (RMSD) computed for the Cα atoms of CCR5 and CXCR4 during the atomistic MD calculations in POPC/CHOL membrane (POPC = 1-palmitoyl-2-oleoyl-sn-glycero-3-phosphatidylcholines, CHOL = cholesterol) on the minima of the CCR5 and CXCR4 homo- and heterodimers identified by CG-MetaD calculations.

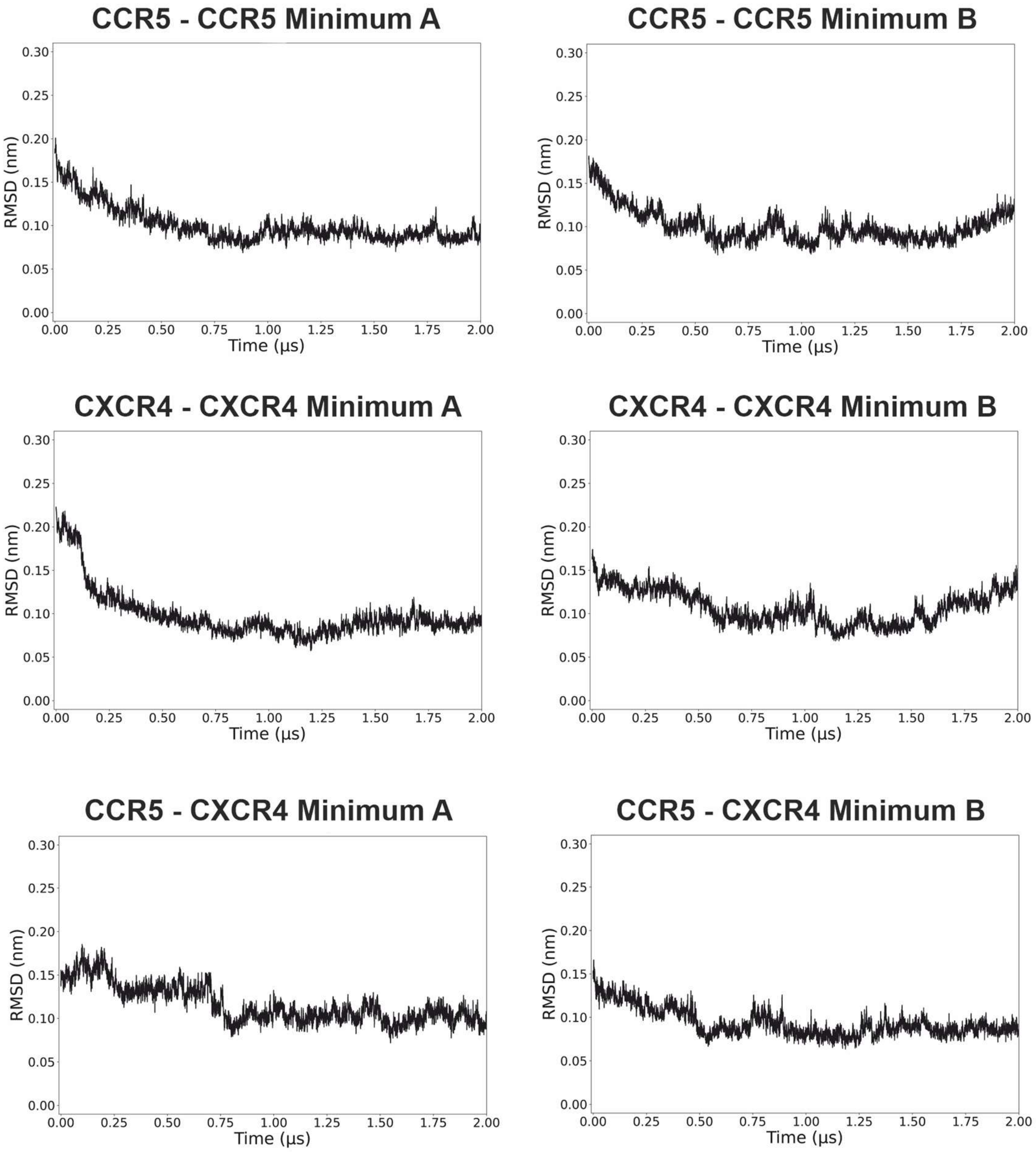

Supplementary Figure 4. **Stability of the identified minima in the plasma membrane model.** Plots of the Root Mean Square Deviation (RMSD) computed for the Cα atoms of CCR5 and CXCR4 during the atomistic MD calculations in plasma membrane on the minima of the CCR5 and CXCR4 homo- and heterodimers identified by CG-MetaD calculations.

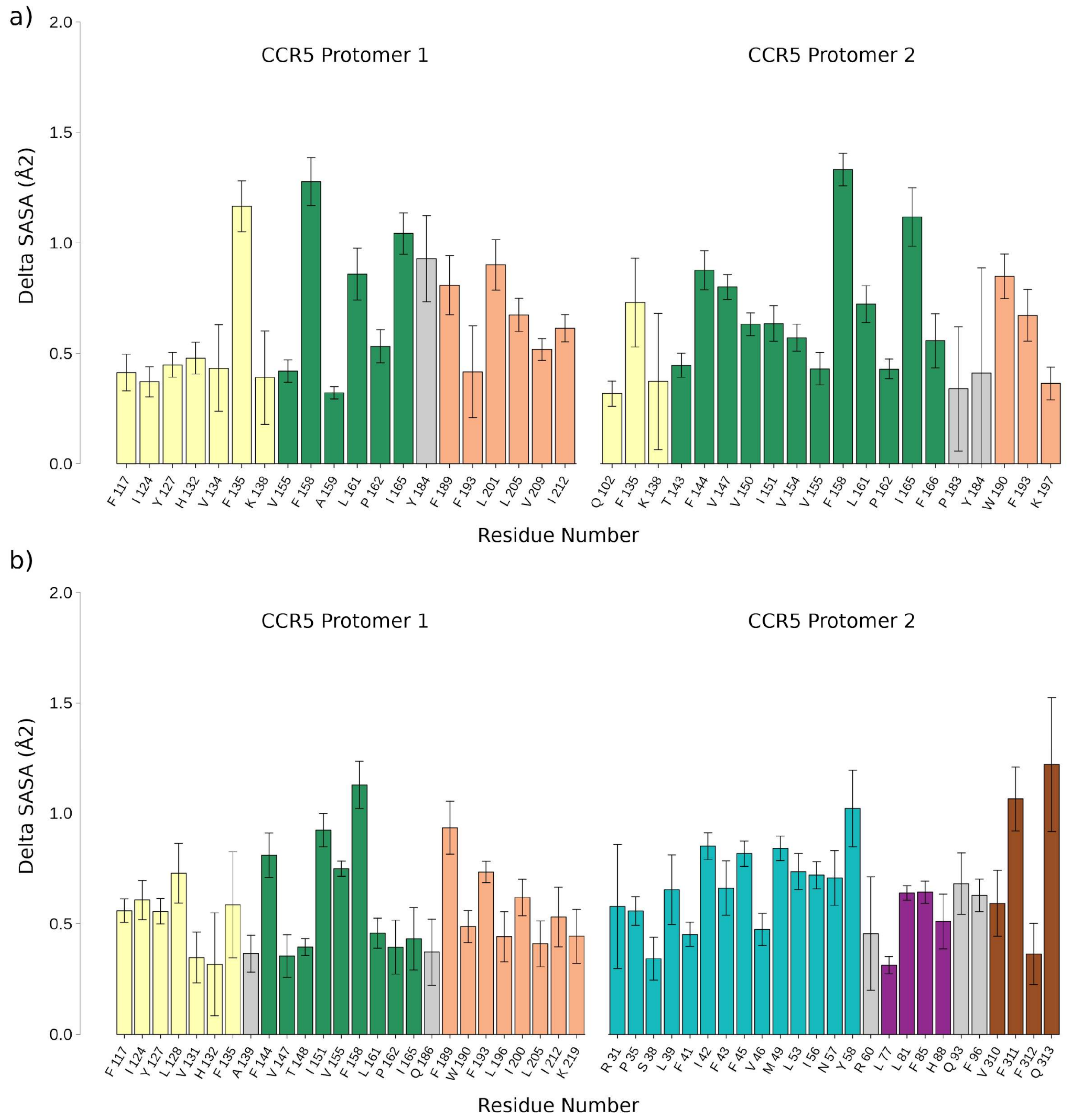

Supplementary Figure 5. **Differences in the homodimeric interfaces of CCR5 minima A and B.** The values of the difference in Solvent Accessible Surface Area (ΔSASA) computed per-residue during 0.5 μs atomistic MD simulations in the POPC/CHOL membrane (POPC = 1-palmitoyl-2-oleoyl-sn-glycero-3-phosphatidylcholines, CHOL = cholesterol) of the CCR5 homodimer structures **a** A and **b** B with respect to the CCR5 monomer state. The higher the ΔSASA value, the more involved are the residues in the binding. Error bars represent standard deviation, n = 10000 frames analyzed for each simulation.

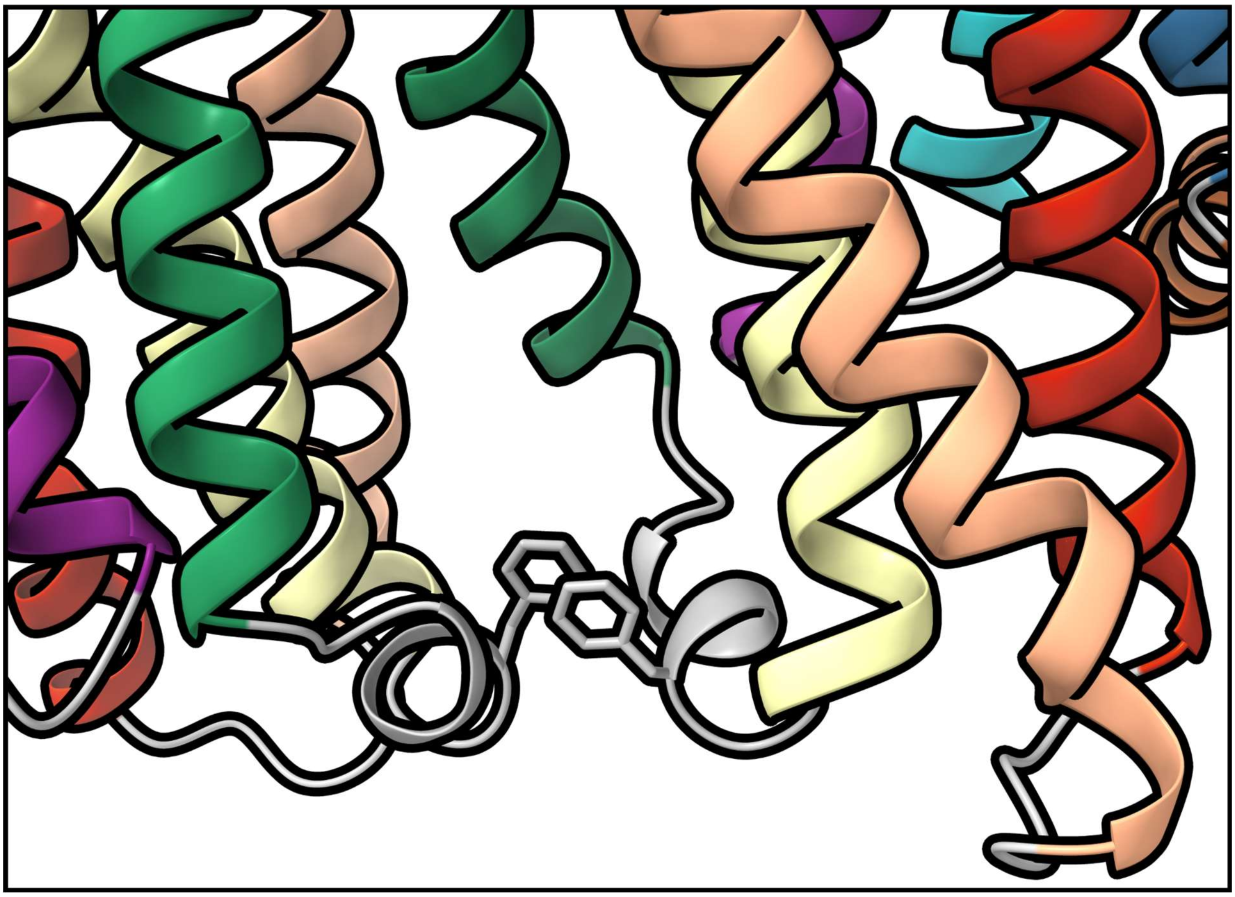

Supplementary Figure 6. **Detail of the CCR5 homodimer.** Representation of the π-stacking interaction engaged by Phe135^{IC2} of facing protomers in the CCR5 homodimer structure A.

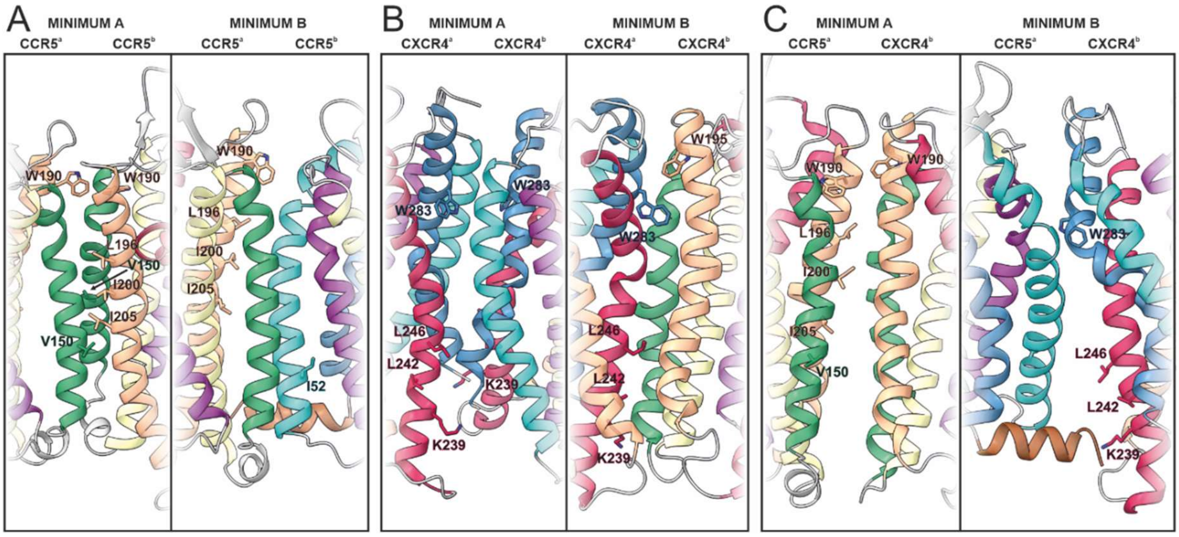

Supplementary Figure 7. D**etail of mutated residues for all dimers.** Detail of some of the residues mutated in Supplementary Tables 4 and 5 at the interfaces of **a** CCR5 and **b** CXCR4 homodimers, and **c** CCR5-CXCR4 heterodimers. The TMs are coloured according to the colour code reported in Fig. 1.

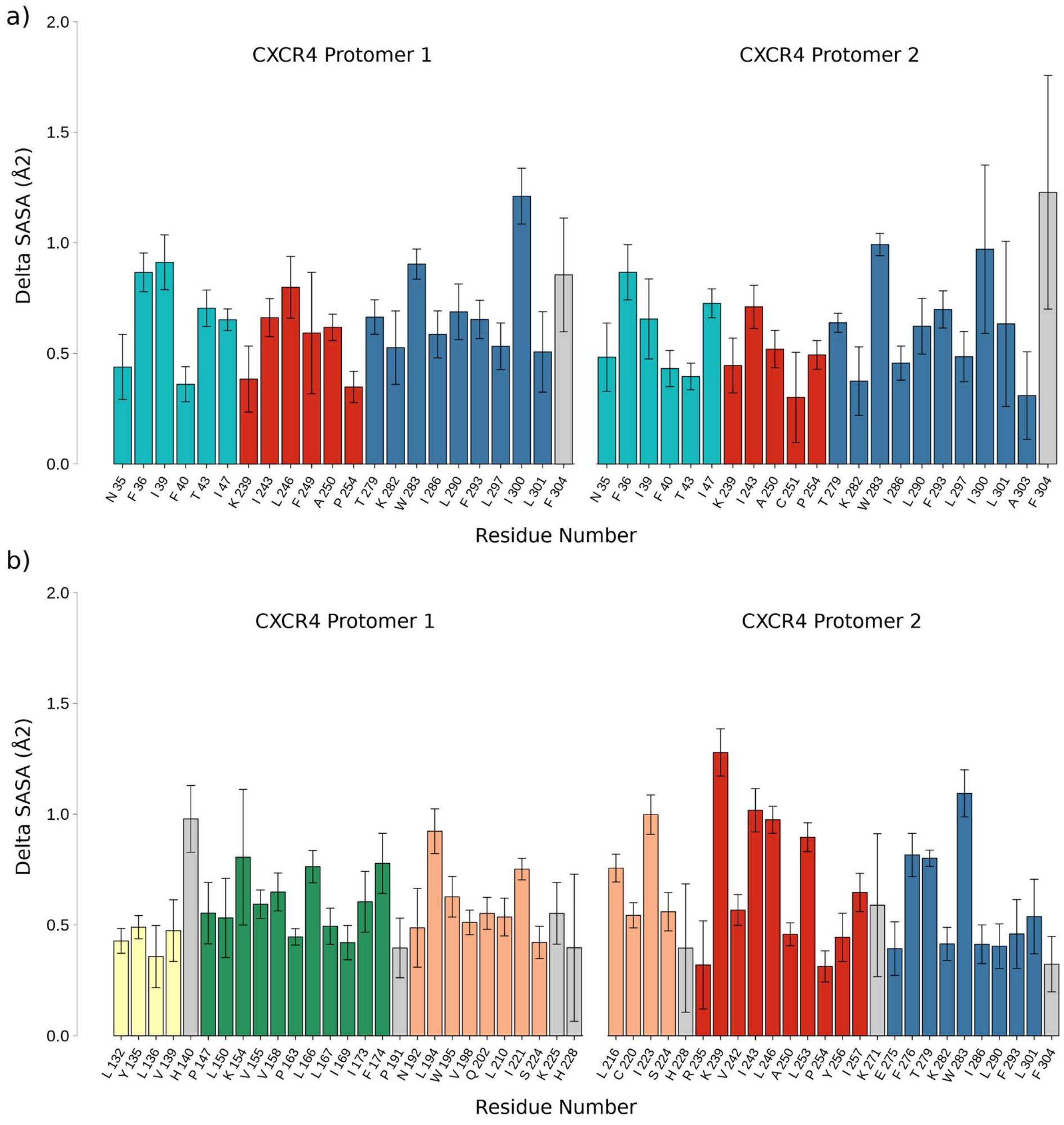

Supplementary Figure 8. **Differences in the homodimeric interfaces of CXCR4 minima A and B.** The values of the difference in Solvent Accessible Surface Area (ΔSASA) computed per-residue during 0.5 μs atomistic MD simulations in the POPC/CHOL membrane (POPC = 1-palmitoyl-2-oleoyl-sn-glycero-3-phosphatidylcholines, CHOL = cholesterol) of the CXCR4 homodimer structures **a** A and **b** B with respect to the CXCR4 monomer state. The higher the ΔSASA value, the more involved are the residues in the binding. Error bars represent standard deviation, n = 10000 frames analyzed for each simulation.

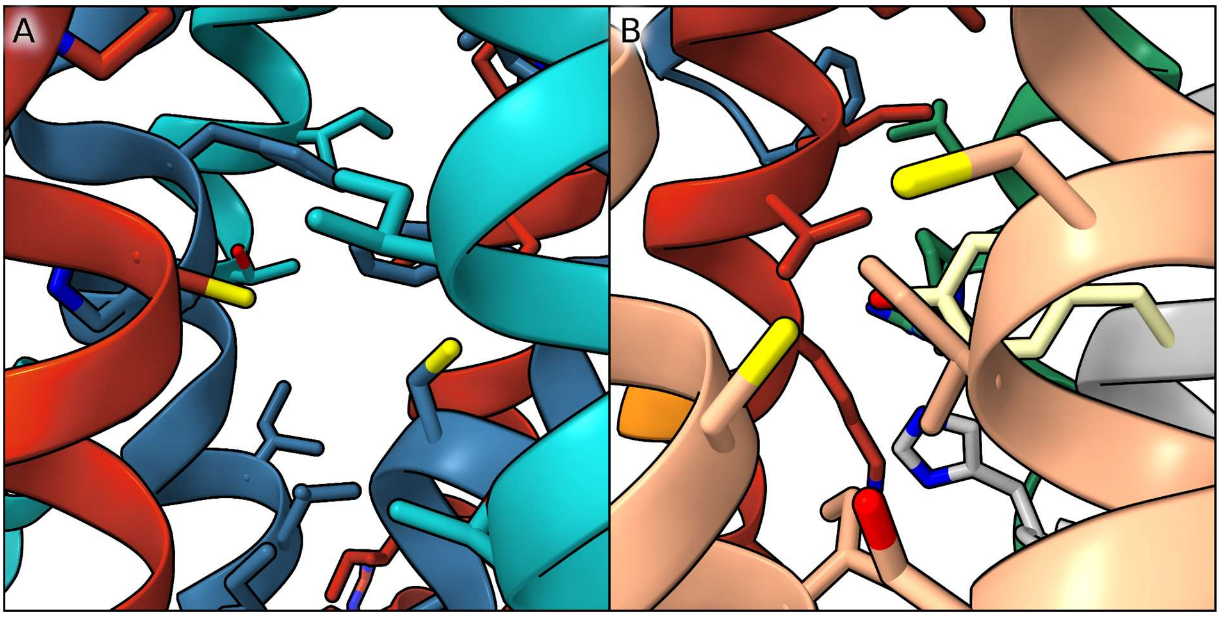

Supplementary Figure 9. **Detail of CXCR4 homodimers.** Representation of the cysteine residues that are placed in a competent position to form a disulphide bridge as found during the atomistic MD calculations on the CXCR4-CXCR4 heterodimer structures **a** A and **b** B.

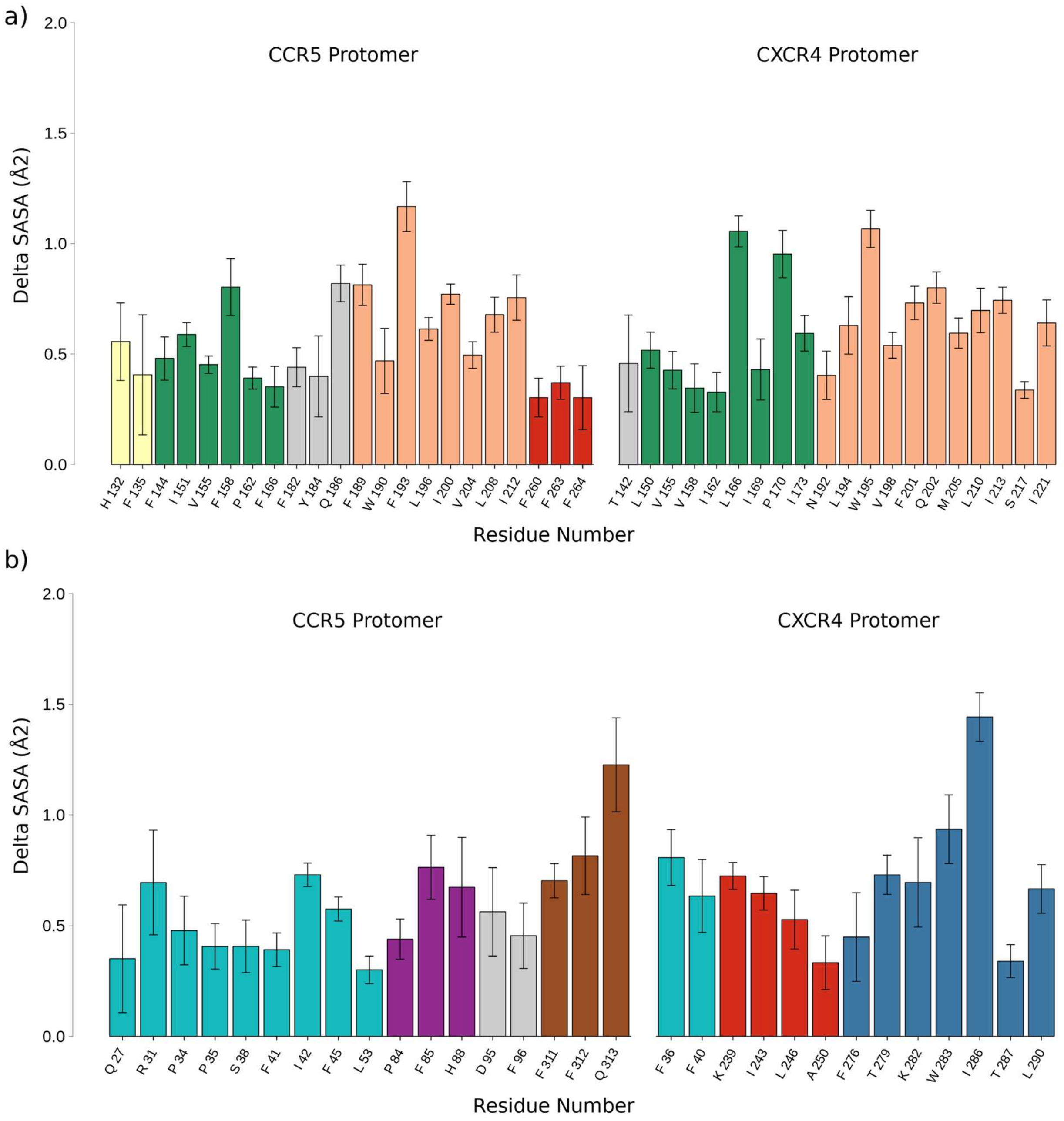

Supplementary Figure 10. **Differences in the dimeric interfaces of CCR5-CXCR4 minima A and B.** The values of the difference in Solvent Accessible Surface Area (ΔSASA) computed per-residue during 0.5 μs atomistic MD simulations in the POPC/CHOL membrane (POPC = 1-palmitoyl-2-oleoyl-sn-glycero-3-phosphatidylcholines, CHOL = cholesterol) of the CCR5-CXCR4 heterodimer structures **a** A and **b** B with respect to the CCR5 and CXCR4 monomer states. The higher the ΔSASA value, the more involved are the residues in the binding. Error bars represent standard deviation, n = 10000 frames analyzed for each simulation.

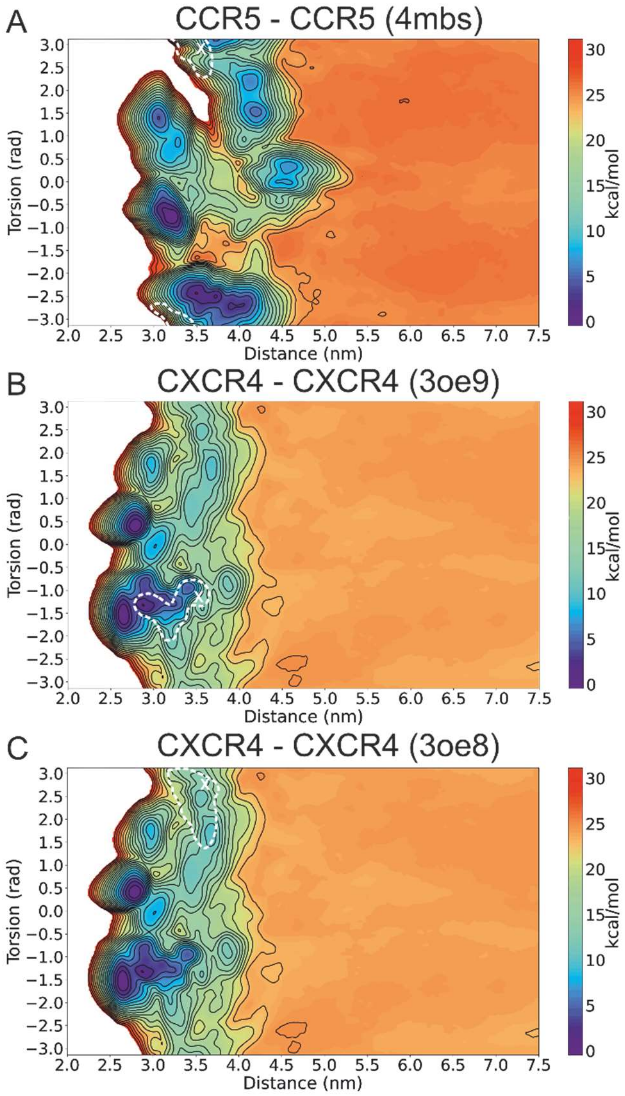

Supplementary Figure 11. **Exploration of the CV space of the CCR5 and CXCR4 homodimers.** The starting points for the CG-MD calculations in a POPC/CHOL membrane model are the Xray conformations. The white dashed lines represent the movement in CV space of **a** the CCR5 dimer starting from PDB ID 4mbs[2], the CXCR4 dimers starting from PDB ID **b** 3oe9[3] and **c** 3oe8[3]. The white X represents the starting positions of the crystallographic structures. In the background, the FES computed for the CCR5 and CXCR4 homodimers via CG-MetaD calculations is represented for the sake of clarity.

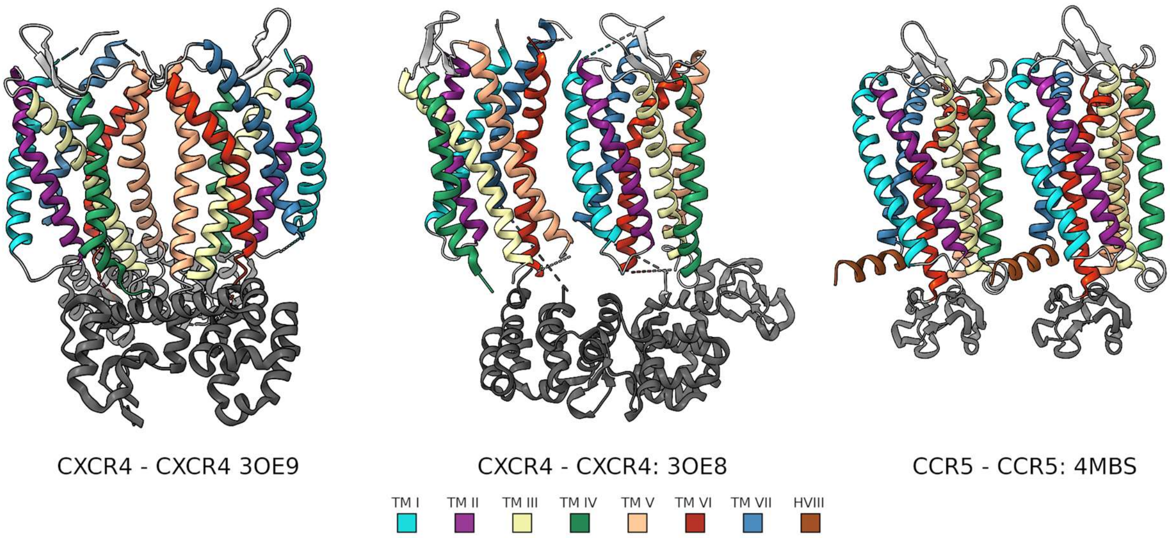

Supplementary Figure 12. **Representation of the X-ray structures of CXCR4 and CCR5.** The receptors dimers of CXCR4 and CCR5 are displayed as coloured cartoons, while the bulky crystallization adjuvant molecules (lysozyme and rubredoxin) as grey cartoon.

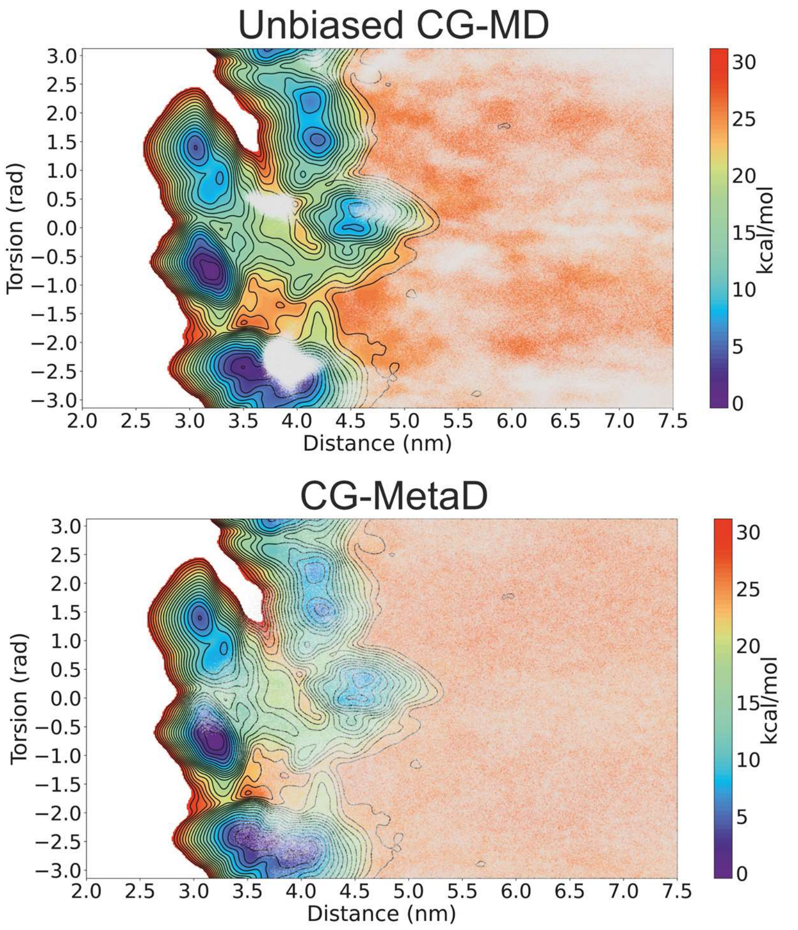

Supplementary Figure 13. **Comparison of sampling power between CG-MD and CG-MetaD.** Phase space exploration represented as light grey spots during 30 µs unbiased CG-MD and CG-MetaD calculations on the CCR5-CCR5 system in the plasma membrane model using Martini 3 force field.

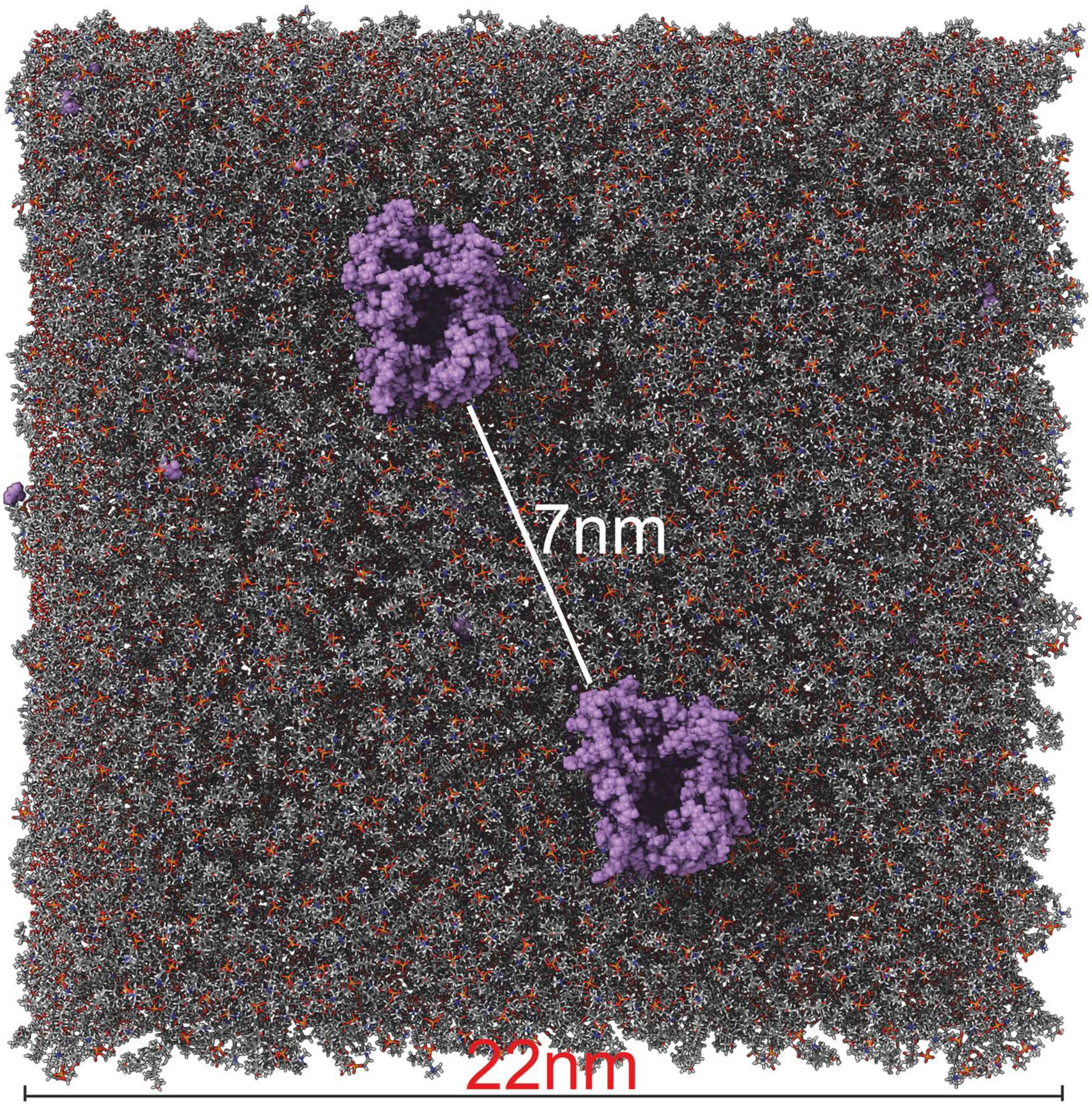

Supplementary Figure 14. **Example of starting conformation.** Representation of chemokine GPCRs in the unbound state at the beginning of the CG-MetaD simulations.

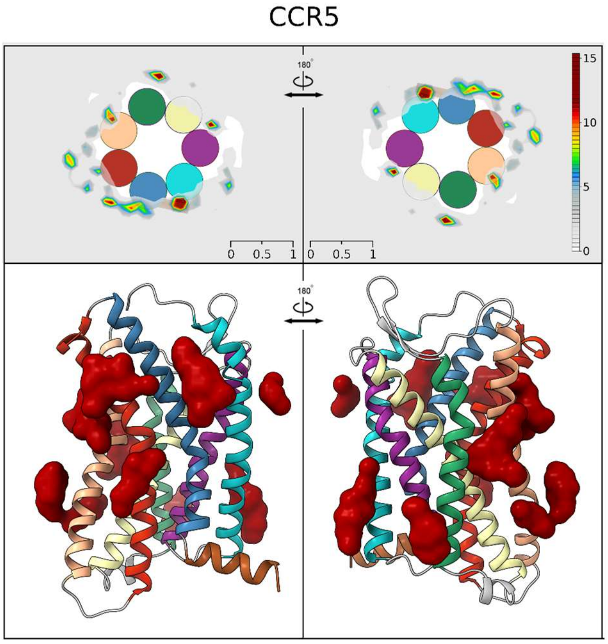

Supplementary Figure 15. **Cholesterol density mapping in CCR5 and CXCR4 monomers.** High-density regions of cholesterol molecules in the CCR5 (left) and CXCR4 (right) monomeric states are represented as 2D extracellular view (top) and in the 3D atomistic structure of the proteins (bottom).

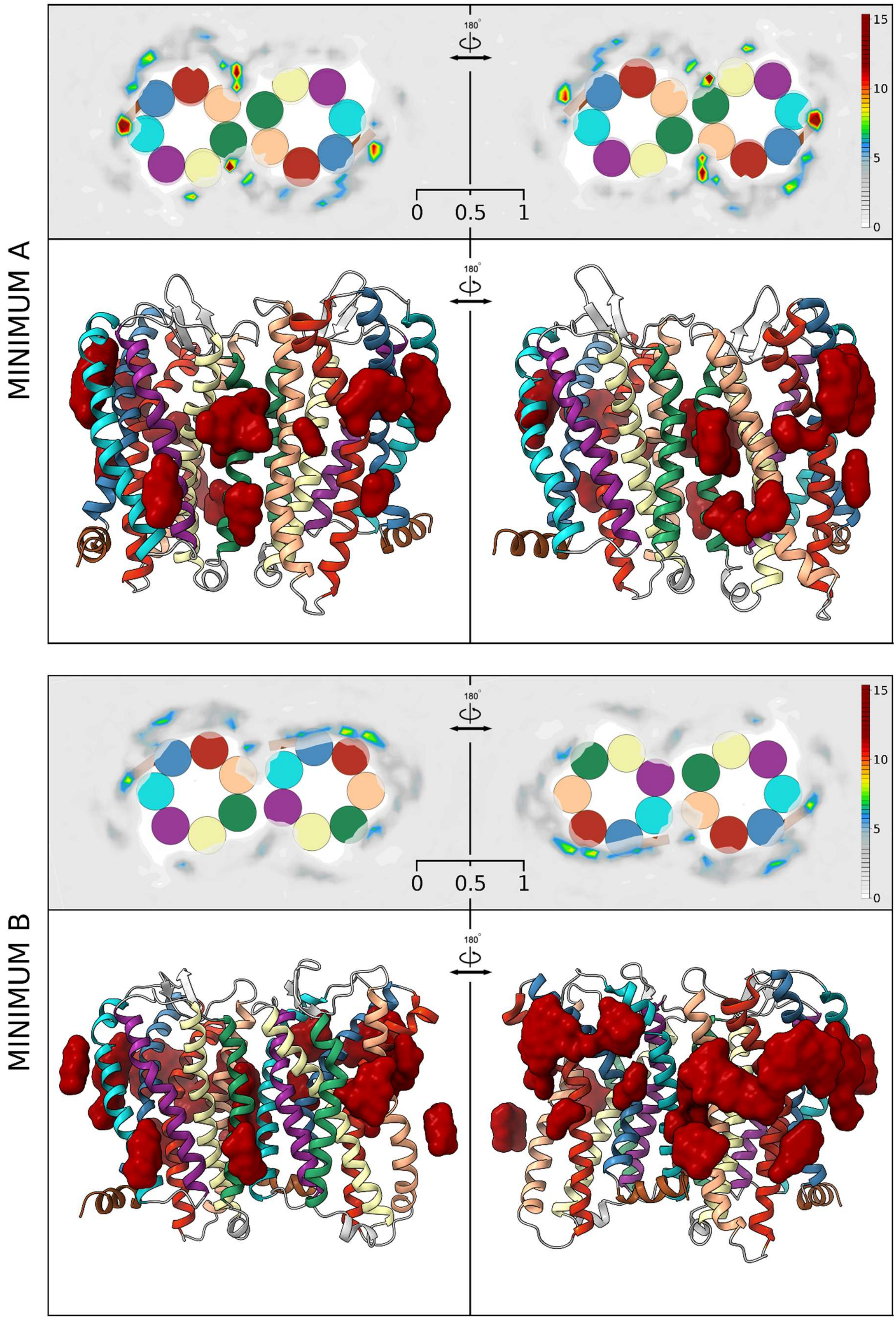

Supplementary Figure 16. **Cholesterol density mapping in CCR5 homodimers.** High-density regions of cholesterol molecules in the CCR5 homodimer structures are represented as 2D extracellular view (top) and in the 3D atomistic structure of the proteins (bottom).

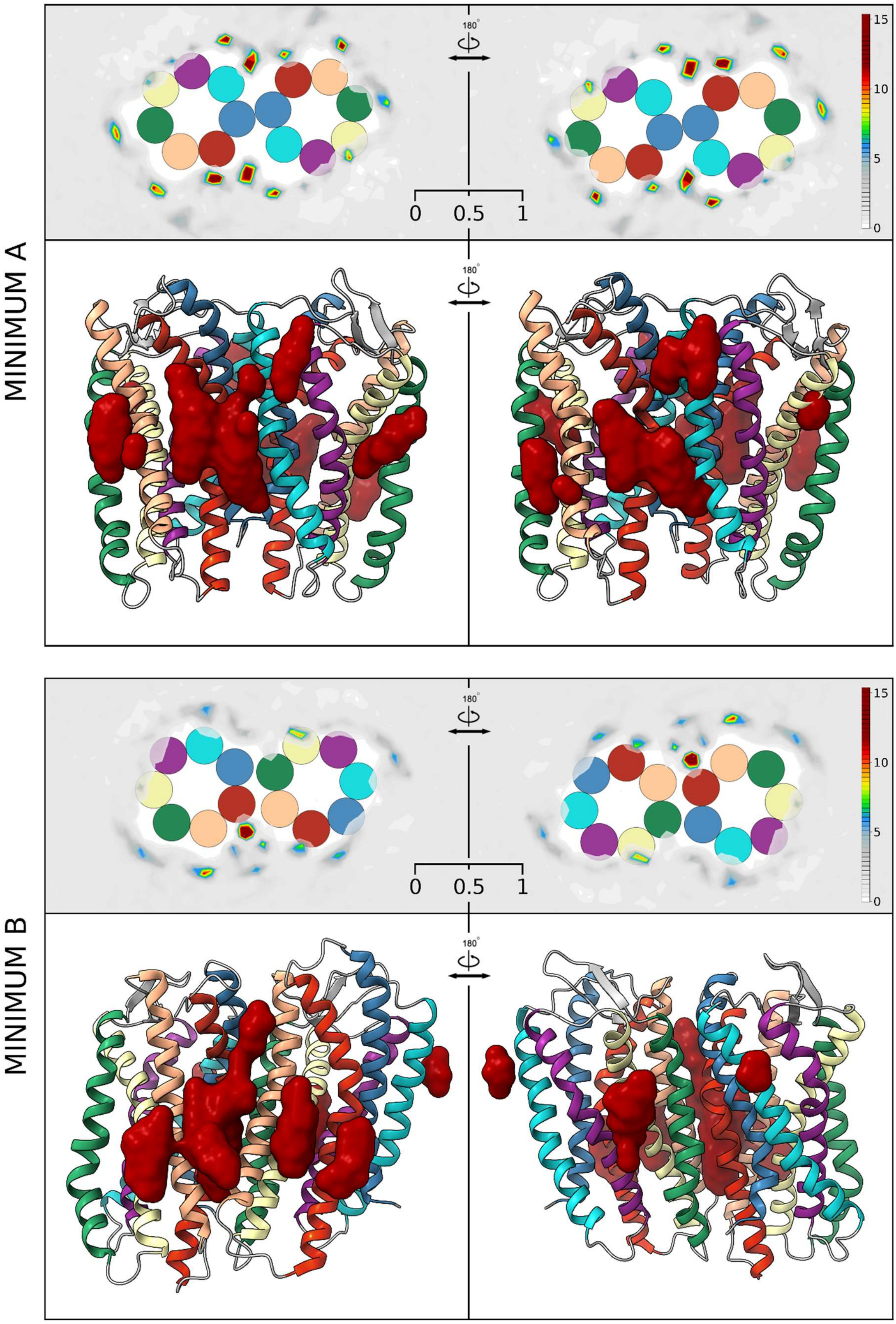

Supplementary Figure 17. **Cholesterol density mapping in CXCR4 homodimers.** High-density regions of cholesterol molecules in the CXCR4 homodimer structures are represented as 2D extracellular view (top) and in the 3D atomistic structure of the proteins (bottom).

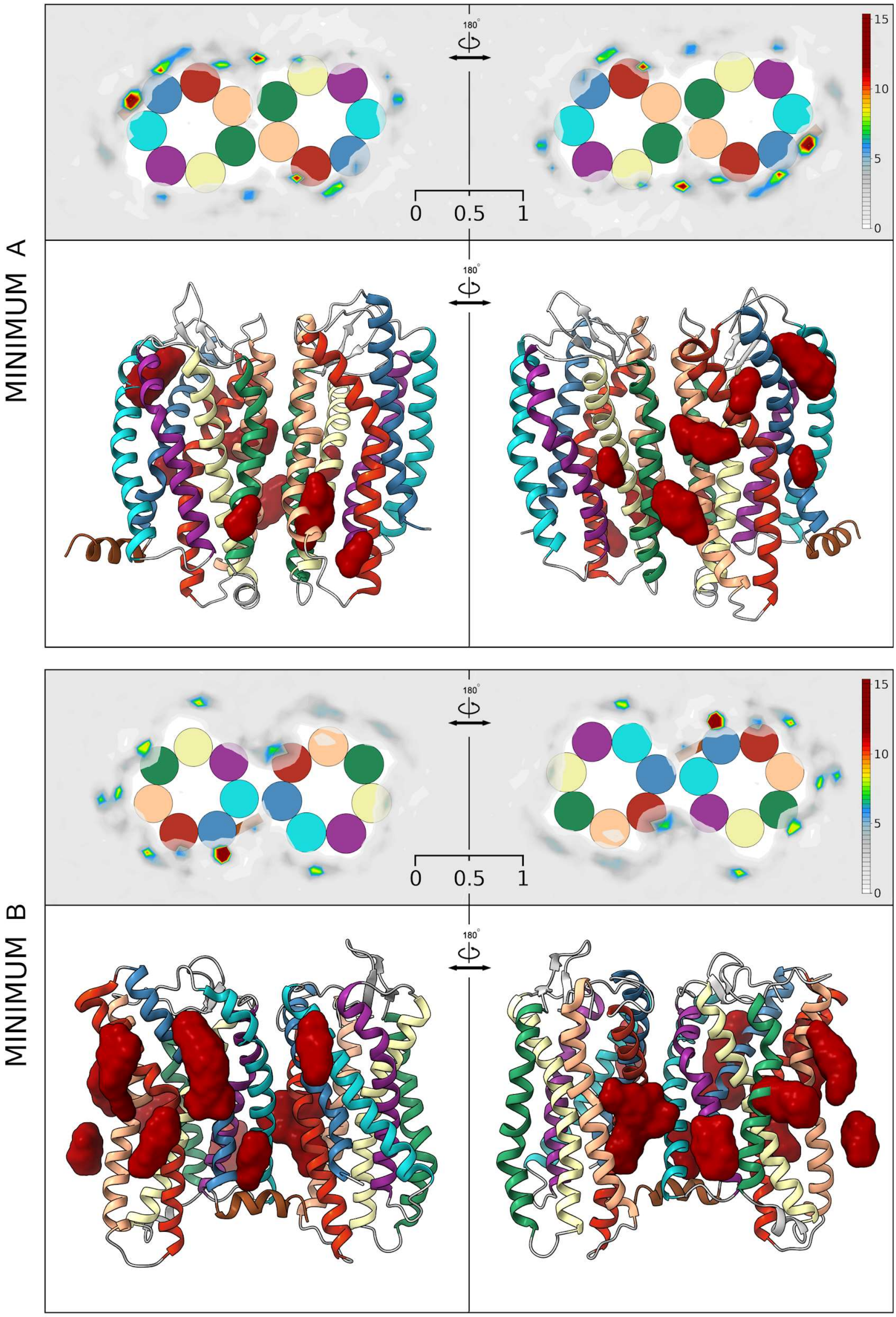

Supplementary Figure 18. **Cholesterol density mapping in CCR5-CXCR4 dimers.** High-density regions of cholesterol molecules in the CCR5-CXCR4 heterodimer structures are represented as 2D extracellular view (top) and in the 3D atomistic structure of the proteins (bottom).

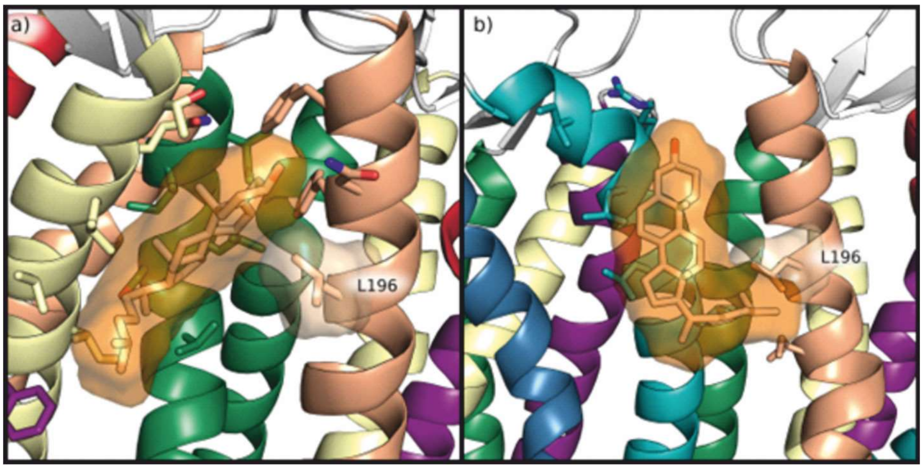

Supplementary Figure 19. **Detail of CCR5 homodimers.** Interaction between L196 and a cholesterol molecule at the binding interface of **a** the symmetric dimer structure A and **b** the asymmetric dimer structure B (b).

Supplementary Discussions

The role of cholesterol in CCR5 and CXCR4 dimerization

The membrane components including cholesterol molecules are known to be able to modulate GPCR activity and dimerization[4–6]. In order to investigate the role of cholesterol molecules in the formation of the CCR5 and CXCR4 dimer complexes, we computed cholesterol density maps around the receptors at the end of the investigated binding process. We note that in our study all the molecules are fully free to diffuse in the membrane, including the cholesterol molecules that were equally distributed in the two layers of the membrane at the beginning of the simulations. Our results show a high density of cholesterol molecules in specific spots of the receptors both in the monomer and dimer states where the GPCRs residues form binding sites for cholesterol (Supplementary Figures 15-19).

Monomeric state - CCR5 shows different cholesterol "hot spots", such as the one formed by TM1-TM2 toward the intracellular side with the residues $F45^{1.47}$, $I52^{1.54}$, $L53^{1.55}$, $I56^{1.58}$, $L70^{2.44}$, $I74^{2.48}$, $L77^{2.51}$ (Supplementary Figure 15, superscripts refer to the Ballesteros-Weinstein numbering scheme[1]), where the side chain of $N57^{1.59}$ interacts with the cholesterol polar head. Another high-density region was found between TM4-TM5, in the transmembrane domain (residues $F113^{3.37}$, $F117^{3.41}$, $V155^{4.52}$, $A159^{4.56}$, $L201^{5.45}$, $L205^{5.49}$). These two helices form a hydrophobic pocket that accommodates a cholesterol molecule, with the polar head interacting with residues $K197^{5.41}$ and $Q194^{5.38}$. Furthermore, a hydrophobic pocket showing cholesterol high density is located toward the extracellular side between TM5-TM6 (residues $L196^{5.40}$, $V199^{5.43}$, $L203^{5.47}$, $V204^{5.48}$, $I253^{6.53}$, $L256^{6.56}$, $F260^{6.60}$, with the cholesterol polar head interacting with $N192^{5.36}$). An additional high cholesterol density region was found between TM1-TM7 (residues $V25^{1.27}$, $I28^{1.30}$, $L32^{1.34}$, $L36^{1.38}$, $V281^{7.37}$ and $T284^{7.40}$) where the presence of a cavity allows the polar head of cholesterol to interact with two glutamine residues in the upper part of TM7 ($Q277^{7.33}$ and $Q280^{7.36}$). Finally, a number of lower density spots were found around TM5-TM6 at the intracellular side of the receptor.

On the other hand, CXCR4 presents a higher cholesterol density at the extracellular side between TM1-TM2. Residues $I39^{1.33}$, $P42^{1.36}$, $F49^{1.43}$, $A95^{2.61}$, $V96^{2.62}$ and $V99^{2.65}$ form a hydrophobic pocket, while $N35^{1.29}$ and $N101^{2.67}$ are two polar residues directly involved in interaction with cholesterol. A further relevant density spot was found between TM2-TM3-TM4, in the middle of the membrane (residues $L86^{2.52}$, $V114^{3.30}$, $I115^{3.31}$, $V118^{3.34}$, $W161^{4.50}$, $L165^{4.54}$) where the polar head of cholesterol can interact with $N119^{3.35}$. Finally, three additional sites were found at the intracellular side of TM5-

TM6-TM7. This region of the receptor is rich in hydrophobic residues able to well accommodate the steroidal scaffold of cholesterol.

Dimeric state - Interestingly, we found that when the protomers are in the dimeric state, they form novel binding sites for cholesterol, alternative to those described for the monomeric state. In detail, Supplementary Figures 16-18 shows the density of cholesterol molecules around the dimer structures representing the lowest energy minima. In almost every dimer state analysed, one or more cholesterol molecules mediate the interaction between protomers. Most of the cholesterol "hot spots", located far from the dimeric interface, are conserved. However, at the dimer interface additional, novel cholesterol binding pockets are formed.

As discussed in the main text, in the CXCR4 dimer structure B (Supplementary Figure 17, asymmetric binding mode) a cholesterol molecule stabilises the dimer complex, binding in between the two protomers (Figure 3 in the main text). Similarly, in both the dimer structures A and B of CCR5 (Supplementary Figure 16), a cholesterol molecule binds to TM4 and TM5 that are involved in the dimerisation interface. These two helices form a hydrophobic pocket made by F113$^{3.37}$, F117$^{3.41}$, V155$^{4.52}$, A159$^{4.56}$, L201$^{5.45}$, L205$^{5.49}$, which accommodates a cholesterol molecule, with its polar head interacting with residues K197$^{5.41}$ and Q194$^{5.38}$. In the CCR5 homodimer structure B (Supplementary Figure 16, asymmetric binding mode), an additional cholesterol molecule interacts with TM1. The presence of such cholesterol molecules contributes to stabilise the dimer structures, however, at variance with CXCR4, in CCR5 they are only partially involved in the binding interface. It is worth noting that the mutation of L196$^{5.40}$, directly involved in the interaction with cholesterol (Supplementary Figure 19), in lysine decreases the dimer formation in favour of the monomeric form, improving the CCR5 affinity for HIV gp120[7]. Based on this evidence, it can be suggested that an exogenous control and stabilisation of the CCR5 dimer structures could be an attractive strategy to achieve an antiviral effect. Finally, while in the CCR5/CXCR4 heterodimer structure A (Supplementary Figure 18, symmetric binding mode) no cholesterol was found at the binding interface, in the dimer structure B (Supplementary Figure 18, asymmetric binding mode) cholesterol binds to TM6 of CXCR4, mediating its interaction with TM1-TM2 of CCR5.

Overall, our findings indicate that cholesterol contributes to the formation and stabilisation of the dimer structures, binding to specific pockets of the receptors. Some of these are peculiar to the receptor dimer structures, while others are also present in the monomer states.

Dimerization mechanism

CCR5 dimer - Three Lowest Energy Paths (LEPs) are found from the monomer to the dimer states of CCR5 (Fig. 5A, red, black, and purple solid lines). Two of them (black and purple) lead to the lowest energy dimer states A and B. Interestingly, the LEP ending in A passes through the metastable state α, with the symmetric binding interface TM1-TM2-H8 (Fig. 5A) and it branches before reaching minimum A. This new path (red solid line) reaches another dimer state β, which has a higher energy value compared to A and B (-17.2 kcal/mol) and an asymmetric binding interface involving TM1^{a}-TM2^{a}-H8^{a}/TM3^{b}-TM4^{b}. The LEP ending in minimum B (Fig. 5A, purple solid line) does not pass through any metastable state, but instead it directly reaches the basin of this minimum.

CXCR4 dimer - A single LEP was identified for the CXCR4 homodimer that from the monomer state splits into two paths reaching the two lowest energy dimers A and B (Fig. 5B). One LEP passes through metastable state δ and then reaches A, whereas the other passes through metastable state ϵ before reaching B (black and purple solid lines, respectively). The metastable state δ shows a symmetric binding interface using TM5-TM6, while the metastable state ϵ is characterised by an asymmetric binding interface formed by TM4^{a}-TM5^{a}-TM6^{a}/TM1^{b}.

CCR5-CXCR4 dimer - The CCR5-CXCR4 heterodimer has a single LEP connecting the monomer state and the dimers A and B (Fig. 5C). This path passes through the metastable state ζ with a binding interface formed by $TM1^a$-$TM7^a$-$H8^a$/$TM5^b$-$TM6^b$. From this state, the system can reach A and B following two separate paths (purple and black solid lines in Fig. 5C, respectively).

Finally, in order to energetically assess the CCR5 and CXCR4 X-ray dimer structures, a total of 480 μs of unbiased CG-MD calculations were performed using these structures as starting state (160 μs per system). We note that during such simulations, the systems only partially explore the free energy landscape (Supplementary Fig. 11). This is due to the slow receptor diffusion in the membrane, the many energy basins to visit and the barriers to cross, which make necessary the employment of enhanced sampling techniques like CG-MetaD for a thorough exploration of the free energy landscape.

Supplementary Methods

Equilibration protocol in CG-MetaD calculations

All the systems were subjected to a multistep equilibration protocol, with the aim of reducing the unfavourable contacts between atoms, however preserving the original protein conformation. This protocol is important in CG simulations, as the membrane model built by the insane algorithm, might not be well equilibrated. First, we performed 10,000 steps of NVT MD simulation (at 300 K) with restraints on protein backbone beads (1000 kJ mol^{-1} nm^{-2}) using a very small timestep of 1.0 fs. This procedure was repeated three times gradually increasing the timestep to 2.0 fs, 4.0 fs and 10.0 fs and keeping the restraints constant. This procedure allows for eliminating bad contacts between the membrane beads and increasing the timestep to 20 fs for the subsequent steps. Then, 20 ns MD was performed, maintaining the restraints at 1000 kJ mol^{-1} nm^{-2} before passing to the NPT ensemble and performing three 5 ns runs in which the restraints are gradually decreased (1000, 500 and 0 kJ mol^{-1} nm^{-2}).

All the following CG-MD simulations were performed in the NPT ensemble at the temperature of 300K. The v-rescale thermostat[8] (coupling constant of 0.1 ps) was used and pressure was set to 1 bar with the semi-isotropic Berendsen barostat[9] (coupling constant of 5.0 ps). LINCS[10] algorithm was used to preserve bonds' lengths. The cut-off method was used to treat long-range electrostatic interactions with the cutoff distance set to 1.1 nm. Short-range repulsive and attractive dispersion interactions were simultaneously described by a Lennard-Jones potential, with a cutoff at 1.1 nm.

Equilibration protocol in atomistic calculations

The CG structures were reverted to atomistic structures that underwent a multistep equilibration protocol. The back-mapping procedure from the CG to the atomistic structures might produce high-energy, unstable conformations that require an adequate preparation before running the production all-atom MD calculations. As a first step, a steepest descent minimisation calculation was performed excluding the non-bonded interactions within membrane and protein atoms[11]. This is done to avoid simulation issues in this stage due to forces generated by overlapping atoms. This step was followed by a steepest descent minimisation calculation that now includes the non-bonded interactions, followed by a series of short NVT MD simulations at increasing timesteps (from 0.2 to 2 fs). During the second minimisation and the MD simulations, restraints with a force constant of 1000 kJ mol^{-1} nm^{-2} were applied to protein backbone atoms and the phospholipid heads. The obtained system was then fully equilibrated with a second longer equilibration protocol schematised in the following table.

Equilibration Step	Timestep (fs)	Number of steps	Restraint force on backbone (kJ mol^{-1} nm^{-2})	Restraint force on lipid polar head (kJ mol^{-1} nm^{-2})
1	1.0	250,000	4000	1000
2	1.0	250,000	2000	1000
3	1.0	250,000	1000	400
4	2.0	250,000	500	200
5	2.0	250,000	200	40
6	2.0	625,000	50	0

All the atomistic MD simulations were performed in the NPT ensemble at the temperature of 300K. The v-rescale thermostat[8] (coupling constant of 0.1 ps) was used and pressure was set to 1 bar with the semi-isotropic Berendsen barostat[9] (coupling constant of 5.0 ps). LINCS[10] algorithm was used to preserve bonds' lengths. The particle mesh Ewald method[12] was used to treat long-range electrostatic interactions with the cutoff distance set to 1.2 nm. A Coulomb and Lennard-Jones potential, with a cut-off at 1.2 nm, were used to treat short-range electrostatic and dispersion interactions, respectively.

Setup of CG-MetaD calculations

Parameters for the CG-MetaD calculations were accurately chosen based on preliminary atomistic and CG MD simulations. In particular, the definition of the MetaD parameters requires particular attention since the CG force fields are less accurate than the atomistic ones, and some simulation parameters could be adequately adapted. As the first step, we performed a CG-MetaD run on the CCR5-CCR5 system, with tentative Gaussian width and height. In this run, we used a value of 0.05 nm for r and 0.1 rad for Ω as Gaussian width and 0.5 kJ/mol as Gaussian height with a pace of 500 steps. This simulation was run as a single walker. After 130 µs several binding and unbinding events were observed with the receptors' secondary structure preserved and the system able to cross even large free-energy barriers separating the diverse energy minima. Therefore, we maintained 0.5 kJ/mol as Gaussian height with a pace of 500 steps in the production runs. As regards Gaussian width, starting from the lowest energy minima states identified in the preliminary CG-MetaD simulation, we performed 10 µs unbiased CG-MD simulations collecting statistics on structural and energetic data in the free-energy wells. The evolution of the r and Ω CVs during such simulations was monitored, and the final values of the Gaussian width for the r and Ω CVs were chosen as half of the standard deviation computed from the values collected for the CVs in the energy minima (0.04 nm for r and 0.06 rad for Ω)[13].

Lowest Energy Paths identification

The Lowest Energy Paths (LEPs) of receptor dimerisation were computed for each system using the data of the Binding Free Energy Surface (BFES) obtained from the CG-MetaD simulations. LEPs were calculated by subdividing the BFES into a bidimensional grid and, starting from one of the energy minima or metastable states, minimising the energy of each

forward step in the grid. A forward step is an increase in the value of protein-protein distance with respect to the previous one along the grid. The grid was obtained by discretising the bidimensional BFES into points spanning from the lowest values of distance and torsional angle sampled (distance 2 nm, torsion $-\pi$) to the highest one (distance 9 nm, torsion π) with a binning equal to one-third of the Gaussian width deposited during CG-MetaD calculations. Minimisation was performed in an iterative way using in-house tools, by repeating the calculation several times with different starting conditions and grid exploration parameters to reach a converged result. As starting conditions, we used different values of distance and torsional angle on the BFES taken from and around the energy basins of each metastable state/energy minimum. As exploration parameters, we employed different values of increase in the protein-protein distance for the forward step and the maximum amount of allowed change in the torsional angle. This approach allowed avoiding over or under-sampling of the grid. Over-sampling would lead to non-continuous LEPs, whereas under-sampling would result in sub-optimal LEPs due to insufficient minimisation of the energy along the path.

Setup of CG-MD calculations with realistic membrane and active complex

Additional CG-MD calculations were performed on both a) the energy minima extracted from the CG-MetaD simulations, and b) the available experimental dimer structures. Equilibration was performed using the same protocol adopted for CG-MetaD simulations (see Methods for details). In case a), for each system (CCR5 and CXCR4 homodimers, CCR5-CXCR4 heterodimer) the receptor structures representing the regions of the FES around each minimum were clustered employing the GROMOS clustering method[14] based on the Cartesian coordinates of the transmembrane backbone beads using a distance cut-off of 0.2 nm. The centroids of the most populated clusters were then embedded in an asymmetric membrane composed of 10 different lipids with various concentrations, mimicking the composition of in vivo cell membrane[15]. The exact composition of this model of bilayer, referred to as plasma membrane model, is reported in Supplementary Table 3A. Each system was then put in a box of size 16x16x12 nm and solvated with CG water and NaCl 0.15 M. Membrane embedding, water solvation and addition of NaCl were performed using the CHARMM-GUI webserver[16]. Each one of the final 6 systems (two minima for each dimer) was simulated in 16 replicas, for a total of 32 replicas per dimer. The simulation time of each replica was 3.5 μs. The same simulation parameters employed in CG-MetaD and the other CG-MD calculations were used. Calculations were performed using GROMACS 2020.6.[17] To investigate the effect of receptor activation and G protein coupling on the dimerisation process, an activated CG model of CCR5 coupled with a Gαβγ heterotrimer was assembled starting from the experimental structure PDB ID 7F1Q [https://doi.org/10.2210/pdb7F1Q/pdb].[18] Furthermore, the agonist Chemokine C-C Motif Ligand 3 (CCL3) peptide was converted to CG description and bound to the activated CCR5 structure using the binding pose reported in 7F1Q. The stability of the CCL3-CCR5-G protein complex, referred to as aCCR5(G), was extensively tested via CG-MD calculations (16 1 μs-long simulations). To avoid detachment of either CCL3 or G protein from CCR5 during the simulations, flat bottom constraints were applied between the receptor and the ligand, the receptor and the Gα and Gβ subunits, and between the Gα and Gβ subunits using GROMACS "Pull Code" parameters. These constraints were modelled to mimic and preserve the quaternary structure reported in 7F1Q[18]. A CCR5 molecule in the CCR5 dimer and in the CCR5-CXCR4 heterodimer in the unbound state was replaced by aCCR5(G). The new systems were prepared and simulated using the procedure reported for the inactive dimers in the plasma membrane model. To ensure that the starting point of each simulation performed with aCCR5(G) was similar to the lowest energy dimeric structure identified from the CG-MetaD calculations, Target MD simulations (TMD) lasting 500 ns were performed using as reference the backbone beads of the minimum structures. In the case of minima B of both aCCR5(G)-CCR5 and aCCR5(G)-CXCR4 systems, TMD were unable to reproduce the quaternary structure of the inactive dimers due to steric clashes between the G protein and the

inactive protomer. Therefore, 500 ns of CV-based steered MD (SMD) calculations were performed to allow the systems to reach the closest CV values to those of energy basins B. Plumed 2.7.1 was employed to perform both TMD and SMD simulations[19]. These conformations were used as starting structures in the unbiased CG-MD simulations.

In case b), CG-MD simulations were performed using as starting poses the experimental dimeric structures PDB IDs 3OE9 [https://doi.org/10.2210/pdb3OE9/pdb][3] and 3OE8 [https://doi.org/10.2210/pdb3OE8/pdb][3] for the CXCR4 homodimer and PDB ID 4MBS [https://doi.org/10.2210/pdb4MBS/pdb][2] for the CCR5 homodimer. The same computational protocol described for a) was employed, performing 160 μs CG-MD calculations using 8 replicas, each lasting 20 μs.

Additional CG-MD and CG-MetaD calculations with realistic membrane and Martini 3

To further prove the robustness of our computational approach in sampling the protein-protein association process, the CCR5-CCR5 system was also simulated using the Martini 3 force field released during the review process of the present article[20,21]. We performed additional CG-MetaD simulations using the starting configuration employed for the original Martini 2 CG-MetaD calculations and the plasma membrane model reported in Supplementary Table 3B. Unbiased CG-MD calculations were also run to compare the sampling power of CG-MetaD to standard MD calculations.

CG-MetaD and CG-MD simulations were performed using the same simulation protocol and settings previously reported. In both cases, 8 walkers/replicas were simulated for 4 μs (32 μs in total) using GROMACS 2020.6[17]. Supplementary Fig. 13 reports the difference in the capability of CG-MD and CG-MetaD to explore the dimerisation phase space.

In silico mutagenesis experiments

The CG structures of dimeric minima A and B identified for CCR5, CXCR4 and CCR5-CXCR4 were backmapped to atomistic level and simulated for 500 ns as described in the Methods section of the main text. Upon a conformational cluster analysis performed on these atomistic structures using the procedure previously described, the most populated centroids of each minimum were provided to the web servers MutaBind2[22] and mCSM-PPI2[23] to assess the influence of several mutations on the stability of the dimers. Two different types of mutagenesis studies were performed: a) introduction of mutations reported in the literature to affect the dimerisation of CCR5[24,25] and CXCR4[26,27] (see Supplementary Table 4); b) Ala scan of all residues placed at less than 8 Å from the binding interface in all the homo and heterodimers (CCR5, CXCR4 and CCR5-CXCR4) (see Supplementary Table 5). In case a), the dimeric PDB structures having IDs 4MBS [https://doi.org/10.2210/pdb4MBS/pdb][2], 3OE8 [https://doi.org/10.2210/pdb3OE8/pdb][3], and 3OE9 [https://doi.org/10.2210/pdb3OE9/pdb][3] also underwent the same mutations. For each mutation reported in Supplementary Tables 4 and 5, the relative binding free-energy difference ΔΔG was computed as the difference in free energy between the wild-type dimer and the mutated one.

Supplementary References

1. Ballesteros, J. A. & Weinstein, H. [19] Integrated methods for the construction of three-dimensional models and computational probing of structure-function relations in G protein-coupled receptors. in Methods in Neurosciences (ed. Sealfon, S. C.) vol. 25 366–428 (Academic Press, 1995).
2. Tan, Q., Zhu, Y., Li, J., Chen, Z., Gye Won Han, Kufareva, I., Li, T., Ma, L., Fenalti, G., Li, J., Zhang, W., Xie, X., Yang, H., Jiang, H., Vadim Cherezov, Liu, H., Stevens, R. C., Zhao, Q. & Wu, B. Structure of the CCR5 Chemokine Receptor–HIV Entry Inhibitor Maraviroc Complex. Science **341**, 1387–1390 (2013). doi: 10.1126/science.1241475
3. Wu, B., Ellen Y.T. Chien, Mol, C. D., Fenalti, G., Liu, W., Vsevolod Katritch, Abagyan, R., Alexei Brooun, Wells, P. G., Bi, F., Hamel, D. J., Kuhn, P., Handel, T. M., Vadim Cherezov & Stevens, R. C. Structures of the CXCR4 Chemokine GPCR with Small-Molecule and Cyclic Peptide Antagonists. Science **330**, 1066–1071 (2010). doi:10.1126/science.1194396
4. Prasanna, X., Chattopadhyay, A. & Sengupta, D. Cholesterol Modulates the Dimer Interface of the B2-Adrenergic Receptor via Cholesterol Occupancy Sites. Biophys. J. **106**, 1290–1300 (2014). doi: 10.1016/j.bpj.2014.02.002
5. Kiriakidi, S., Kolocouris, A., Liapakis, G., Ikram, S., Durdagi, S. & Mavromoustakos, T. Effects of Cholesterol on GPCR Function: Insights from Computational and Experimental Studies. In: Rosenhouse-Dantsker, A., Bukiya, A. (eds) Direct Mechanisms in Cholesterol Modulation of Protein Function. Advances in Experimental Medicine and Biology, 1135. Springer, Cham. (2019). doi: 10.1007/978-3-030-14265-0_5
6. Pluhackova, K., Gahbauer, S., Kranz, F., Wassenaar, T. A. & Bo, R. A. Dynamic Cholesterol-Conditioned Dimerization of the G Protein Coupled Chemokine Receptor Type 4. PLoS Comput. Biol. 1–25 (2016). doi: 10.1371/journal.pcbi.1005169
7. Colin, P., Zhou, Z., Staropoli, I., García-Pérez, J., Gasser, R., Armani-Tourret, M., Yann Benureau, Nuria González, Jin, J., Bridgette Janine Connell, Raymond, S., Delobel, P., Izopet, J., Hugues Lortat-Jacob, José Alcami, Arenzana-Seisdedos, F., Brelot, A. & Lagane, B. CCR5 structural plasticity shapes HIV-1 phenotypic properties. PLoS Path. **14**, e1007432–e1007432 (2018). doi: 10.1371/journal.ppat.1007432
8. Bussi, G., Donadio, D. & Parrinello, M. Canonical sampling through velocity rescaling. J. Chem. Phys. **126**, (2007). doi: 10.1063/1.2408420
9. Berendsen, H. J. C., Postma, J. P. M., van Gunsteren, W. F., DiNola, A. & Haak, J. R. Molecular Dynamics with Coupling to an External Bath. J. Chem. Phys. **81**, 8, 3684–3690 (1984). doi: 10.1063/1.448118
10. Hess, B., Bekker, H., Berendsen, H. J. C. & Fraaije, J. G. E. M. LINCS: A Linear Constraint Solver for Molecular Simulations. J. Comput. Chem. **18**, 12, 1463–1472 (1998). doi: 10.1002/(SICI)1096-987X(199709)18:12<1463::AID-JCC4>3.0.CO;2-H
11. Wassenaar, T. A., Pluhackova, K., Böckmann, R. A., Marrink, S. J. & Tieleman, D. P. Going Backward: A Flexible Geometric Approach to Reverse Transformation from Coarse Grained to Atomistic Models. J. Chem. Theory Comput. **10**, 2, 676–690 (2014). doi: 10.1021/ct400617g
12. Essmann, U., Perera, L., Berkowitz, M. L., Darden, T., Lee, H. & Pedersen, L. G. A Smooth Particle Mesh Ewald Method. J. Chem. Phys. **103**, 8577–8593 (1995). doi: 10.1063/1.470117
13. Bussi, G. & Branduardi, D. Free-Energy Calculations with Metadynamics: Theory and Practice. Rev. Comput. Chem. **28**, 1–49 (2015). doi: 10.1002/9781118889886.ch1
14. Abraham, M., Murtola, T. J., Schulz, R., Szilárd Páll, Smith, J. C., Hess, B. & Lindahl, E. GROMACS: High performance molecular simulations through multi-level parallelism from laptops to supercomputers. SoftwareX **1-2**, 19–25 (2015). doi: 10.1016/j.softx.2015.06.001
15. Song, W., Yen, H.-Y., Robinson, C. V. & Sansom, M. S. P. State-dependent Lipid Interactions with the A2a

Receptor Revealed by MD Simulations Using In Vivo-Mimetic Membranes. Structure **27**, 392-403.e3 (2019). doi: 10.1016/j.str.2018.10.024
16. Hsu, P.-C., Marlon, B., Jefferies, D., Paulo, Lee, J., Patel, D. S., Marrink, S. J., Qi, Y., Khalid, S. & Im, W. CHARMM-GUI Martini Maker for modeling and simulation of complex bacterial membranes with lipopolysaccharides. J. Comput. Chem. **38**, 2354–2363 (2017). doi: 10.1002/jcc.24895
17. Kohnke, B., Kutzner, C. & Grubmüller, H. A GPU-Accelerated Fast Multipole Method for GROMACS: Performance and Accuracy. J. Chem. Theory Comput. **16**, 6938–6949 (2020). doi: 10.1021/acs.jctc.0c00744
18. Zhang, H., Chen, K., Tan, Q., Shao, Q., Han, S., Zhang, C., Yi, C., Chu, X., Zhu, Y., Xu, Y., Zhao, Q. & Wu, B. Structural basis for chemokine recognition and receptor activation of chemokine receptor CCR5. Nat. Commun. **12**, 4151 (2021). doi: 10.1038/s41467-021-24438-5
19. Tribello, G. A., Bonomi, M., Branduardi, D., Camilloni, C. & Bussi, G. PLUMED 2: New feathers for an old bird. Comput. Phys. Commun. **185**, 604–613 (2014). doi: 10.1016/j.cpc.2013.09.018
20. Souza, P. C. T., Alessandri, R., Barnoud, J., Thallmair, S., Faustino, I., Grünewald, F., Patmanidis, I., Abdizadeh, H., Bruininks, B. M. H., Wassenaar, T. A., Kroon, P. C., Melcr, J., Nieto, V., Corradi, V., Khan, H. M., Domański, J., Javanainen, M., Martinez-Seara, H., Reuter, N. & Best, R. B. Martini 3: a general purpose force field for coarse-grained molecular dynamics. Nat. Methods **18**, 382–388 (2021). doi: 10.1038/s41592-021-01098-3
21. Borges-Araújo, L., Borges-Araújo, A., Ozturk, T., Ramirez-Echemendia, D. P., Balázs Fábián, Carpenter, T. S., Thallmair, S., Barnoud, J., Ingólfsson, H. I., Hummer, G., D. Peter Tieleman, Marrink, S. J., Paulo & Melo, M. N. Martini 3 Coarse-Grained Force Field for cholesterol. Preprint at https://doi.org/10.26434/chemrxiv-2023-lh7bq (2023).
22. Zhang, N., Chen, Y., Lu, H., Zhao, F., Roberto Vera Alvarez, Goncearenco, A., Panchenko, A. R. & Li, M. MutaBind2: Predicting the Impacts of Single and Multiple Mutations on Protein-Protein Interactions. iScience **23**, 100939–100939 (2020). doi: 10.1016/j.isci.2020.100939
23. Rodrigues, C. H. M., Myung, Y., Pires, D. E. V. & Ascher, D. B. mCSM-PPI2: predicting the effects of mutations on protein–protein interactions. Nucleic Acids Res.**47**, W338–W344 (2019). doi: 10.1093/nar/gkz383
24. Colin, P., Zhou, Z., Staropoli, I., García-Pérez, J., Gasser, R., Armani-Tourret, M., Yann Benureau, Nuria González, Jin, J., Bridgette Janine Connell, Raymond, S., Delobel, P., Izopet, J., Hugues Lortat-Jacob, José Alcami, Arenzana-Seisdedos, F., Brelot, A. & Lagane, B. CCR5 structural plasticity shapes HIV-1 phenotypic properties. PLoS Pathog. **14**, e1007432–e1007432 (2018). doi: 10.1371/journal.ppat.1007432
25. Hernanz-Falcón, P., José Miguel Rodríguez-Frade, Serrano, A., Juan, D., Antonio del Sol, Soriano, S. F., Roncal, F., Gómez, L., Valencia, A., Martínez-A, C. & Mellado, M. Identification of amino acid residues crucial for chemokine receptor dimerization. Nat. Immunol. **5**, 216–223 (2004). doi: 10.1038/ni1027
26. Martínez-Muñoz, L., José Miguel Rodríguez-Frade, Barroso, R., Oscar, C., Torreno-Pina, J. A., Santiago, C., Manzo, C., Lucas, P., García-Cuesta, E. M., Gutierrez, E., Barrio, L., Vargas, J., Cascio, G., Carrasco, Y. R., Sánchez-Madrid, F., Garcia-Parajo, M. F. & Mellado, M. Separating Actin-Dependent Chemokine Receptor Nanoclustering from Dimerization Indicates a Role for Clustering in CXCR4 Signaling and Function. Mol. Cell **70**, 106-119.e10 (2018). doi: 10.1016/j.molcel.2018.02.034
27. Işbilir, A., Möller, J., Arimont, M., Vladimir Bobkov, Perpiñá-Viciano, C., Hoffmann, C., Inoue, A., Raimond Heukers, Chris de Graaf, Smit, M. J., Annibale, P. & Lohse, M. J. Advanced fluorescence microscopy reveals disruption of dynamic CXCR4 dimerization by subpocket-specific inverse agonists. Proc. Natl. Acad. Sci. U.S.A. **117**, 29144–29154 (2020). doi: 10.1073/pnas.2013319117
