# Supplementary material for: Structural basis of dimerization of chemokine receptors CCR5 and CXCR4
Source: Nat Commun. 2023 Oct 13;14:6439. doi: 10.1038/s41467-023-42082-z (PMC10575954; doi:10.1038/s41467-023-42082-z)
Supplement: Supplementary file 3 — Description of Additional Supplementary Files [file 41467_2023_42082_MOESM3_ESM.pdf]

File name: Supplementary Movie 1
Description: Movie showing the dimerization mechanism for each system as captured by the CG-MetaD calculations.
