# Supplementary material for: Structural basis of dimerization of chemokine receptors CCR5 and CXCR4
Source: Nat Commun. 2023 Oct 13;14:6439. doi: 10.1038/s41467-023-42082-z (PMC10575954; doi:10.1038/s41467-023-42082-z)
Supplement: Supplementary file 5 — Reporting Summary [file 41467_2023_42082_MOESM5_ESM.pdf]

nature portfolio

Corresponding author(s): Vittorio Limongelli

Last updated by author(s): Sep 11, 2023

Reporting Summary

Nature Portfolio wishes to improve the reproducibility of the work that we publish. This form provides structure for consistency and transparency in reporting. For further information on Nature Portfolio policies, see our Editorial Policies and the Editorial Policy Checklist.

Statistics

For all statistical analyses, confirm that the following items are present in the figure legend, table legend, main text, or Methods section.

n/a	Confirmed	
☐	☒	The exact sample size (*n*) for each experimental group/condition, given as a discrete number and unit of measurement
☐	☒	A statement on whether measurements were taken from distinct samples or whether the same sample was measured repeatedly
☒	☐	The statistical test(s) used AND whether they are one- or two-sided<br>*Only common tests should be described solely by name; describe more complex techniques in the Methods section.*
☒	☐	A description of all covariates tested
☐	☒	A description of any assumptions or corrections, such as tests of normality and adjustment for multiple comparisons
☐	☒	A full description of the statistical parameters including central tendency (e.g. means) or other basic estimates (e.g. regression coefficient) AND variation (e.g. standard deviation) or associated estimates of uncertainty (e.g. confidence intervals)
☒	☐	For null hypothesis testing, the test statistic (e.g. *F*, *t*, *r*) with confidence intervals, effect sizes, degrees of freedom and *P* value noted<br>*Give P values as exact values whenever suitable.*
☒	☐	For Bayesian analysis, information on the choice of priors and Markov chain Monte Carlo settings
☒	☐	For hierarchical and complex designs, identification of the appropriate level for tests and full reporting of outcomes
☒	☐	Estimates of effect sizes (e.g. Cohen's *d*, Pearson's *r*), indicating how they were calculated

Our web collection on statistics for biologists contains articles on many of the points above.

Software and code

Policy information about availability of computer code

Data collection	Gromacs v5.1 and 2020.6, Plumed 2.3 and 2.7.
Data analysis	Gromacs v5.1 and 2020.6, POPSCOMP server, PISA server, UCSF Chimera X, POVME, Pymol, R and Python 2.7/3 scripts

For manuscripts utilizing custom algorithms or software that are central to the research but not yet described in published literature, software must be made available to editors and reviewers. We strongly encourage code deposition in a community repository (e.g. GitHub). See the Nature Portfolio guidelines for submitting code & software for further information.

Data

Policy information about availability of data

All manuscripts must include a data availability statement. This statement should provide the following information, where applicable:

- Accession codes, unique identifiers, or web links for publicly available datasets
- A description of any restrictions on data availability
- For clinical datasets or third party data, please ensure that the statement adheres to our policy

The identified dimeric structures of CCR5, CXCR4 and CXCR4-CCR5 are available as PDB (Protein Data Bank) files in the Supplementary Materials, at www.pdbdb.com and in the Zenodo database under accession code 10.5281/zenodo.8337056 [https://doi.org/10.5281/zenodo.8337056].
All data generated in this study have been deposited in the Zenodo database under accession code 10.5281/zenodo.8337056 [https://doi.org/10.5281/zenodo.8337056].

Input files and force fields parameters are included. Source data for each figure of both this article and its supplementary information are provided with the paper as Supplementary Information file and in Zenodo.
The CG-MetaD protocol employed in this work is available on PLUMED-NEST under project id plumID:23.014 (https://www.plumed-nest.org/eggs/23/014/).
Python code for evaluation of the Lowest Energy Path (LEP) is available on Github (https://github.com/DarnosAeth/LEPextractor).
Backmapping scripts from CG to atomistic structures and all PLUMED input files are included in the Zenodo database under accession code 10.5281/zenodo.8337056 [https://doi.org/10.5281/zenodo.8337056].
Other analysis tools written in R or Python not included in the dataset are available from the corresponding author on reasonable request.

Human research participants

Policy information about studies involving human research participants and Sex and Gender in Research.

Reporting on sex and gender	N/A
Population characteristics	N/A
Recruitment	N/A
Ethics oversight	N/A

Note that full information on the approval of the study protocol must also be provided in the manuscript.

Field-specific reporting

Please select the one below that is the best fit for your research. If you are not sure, read the appropriate sections before making your selection.

☒ Life sciences ☐ Behavioural & social sciences ☐ Ecological, evolutionary & environmental sciences

For a reference copy of the document with all sections, see nature.com/documents/nr-reporting-summary-flat.pdf

Life sciences study design

All studies must disclose on these points even when the disclosure is negative.

Sample size	MetaDynamics calculations were performed until convergence was achieved. The length of the simulations is 1.4 ms for CCR5-CCR5, 2.5 ms for CXCR4-CXCR4, 1.6 ms for CCR5-CXCR4. Coarse-Grained Molecular Dynamics (CG-MD) calculations for refinement of the lowest energy states lasted 50 µs. Sample size for additional simulations was decided based on the structural stability of the simulated protein. The Root Mean Square Deviation (RMSD), describing the stability of the secondary, tertiary and quaternary structures of the proteins, was monitored during the calculations. RMSD was computed on each protomer and the dimer. Simulations were stopped only after checking that RMSD was stable for at least 100 ns (atomistic models) or 500 ns (coarse grained models). In some cases, and depending on the computational resources available, calculations were continued even when the RMSD was stable to ensure that the structures were indeed trapped in a metastable state. These criteria have been also applied to the following calculations. Atomistic MD simulations of the lowest energy, back-mapped structures lasted 0.5 or 2 µs per dimer. Additional CG-MD calculations performed using different membrane models or different conformational states of the proteins lasted 3 or 3.5 µs per dimer and were replicated 8/16 times. CG-MD calculations run on the experimentally resolved dimers lasted 20 µs and were replicated 8 times. The amount of replica was chosen as to ensure that our data were statistically significant.
Data exclusions	No data were excluded
Replication	MetaDynamics calculations were not replicated due to the nature of the technique, which allows to accurately estimate the energy landscape of a phenomenon by promoting multiple occurrences of the same event. CG and atomistic MD simulations performed on the lowest energy states were not replicated since the systems were stable in the allotted simulation time. CG-MD calculations performed using different membrane models or different conformational states were replicated 8/16 times. CG-MD calculations run on the experimentally resolved dimers were replicated 8 times.
Randomization	No randomization method was employed in this study. Data is generated as time-dependent sequences, and this feature is important to assess the evolution of protein structures, the dimerization mechanism, and convergence of calculation. Therefore, each trajectory/replica was analysed independently and in its entirety to obtain this information. When statistical data were collected from all replicas of a system, frames were pooled together and analysed without considering the trajectory of origin and their time-dependency. In these cases, frames were indeed mixed up and scrambled. Recognizing the origin trajectory of each frame, or time-correlation features, was virtually impossible. However, this is not the result of an actual randomization approach, but the natural result of techniques like clusterization methods where the time evolution of a simulation is not relevant.
Blinding	Blinding methods were not employed in this study. Due to the nature of our calculations, as also reported in the "Randomization" tab, analyzing each trajectory individually is essential. Trajectories cannot be mixed and their content or origin cannot be masked, because it would invalidate the analysis or make it devoid of significance. As an example, it would make no sense to mix frames coming from different systems or obtain through different techniques, since their behaviour is naturally different. Knowledge of input data and simulation conditions is also important to assess the quality of the trajectories and their results.

Reporting for specific materials, systems and methods

We require information from authors about some types of materials, experimental systems and methods used in many studies. Here, indicate whether each material, system or method listed is relevant to your study. If you are not sure if a list item applies to your research, read the appropriate section before selecting a response.

Materials & experimental systems

n/a	Involved in the study
☒	☐ Antibodies
☒	☐ Eukaryotic cell lines
☒	☐ Palaeontology and archaeology
☒	☐ Animals and other organisms
☒	☐ Clinical data
☒	☐ Dual use research of concern

Methods

n/a	Involved in the study
☒	☐ ChIP-seq
☒	☐ Flow cytometry
☒	☐ MRI-based neuroimaging
